# Insight into Cytotoxic Potential of *Erica spiculifolia* Salisb (Balkan Heath)

**DOI:** 10.3390/plants14193063

**Published:** 2025-10-03

**Authors:** Reneta Gevrenova, Rositsa Mihaylova, Nikolay Bebrivenski, Georgi Momekov, Dimitrina Zheleva-Dimitrova

**Affiliations:** 1 Department of Pharmacognosy, Faculty of Pharmacy, Medical University of Sofia, 1000 Sofia, Bulgaria; dzheleva@pharmfac.mu-sofia.bg; 2 Department of Pharmacology, Pharmacotherapy and Toxicology, Faculty of Pharmacy, Medical University of Sofia, 1000 Sofia, Bulgaria; rmihaylova@pharmfac.mu-sofia.bg (R.M.); nikbebsl@gmail.com (N.B.); gmomekov@pharmfac.mu-sofia.bg (G.M.)

**Keywords:** *Erica spiculifolia*, UHPLC-HRMS, triterpene acids, cytotoxicity

## Abstract

*Erica spiculifolia* Salisb. (Balkan heath) is an evergreen shrub growing in the mountain shrublands of Eastern Europe. *E. spiculifolia* was used as a diuretic, anti-inflammatory, and antioxidant herbal remedy. The present study aims to conduct an evaluation of the phytochemical composition and antitumor activity of the methanol–aqueous extract from *E. spiculifolia* aerial parts to explore its potential in cancer treatment. Overall, a total of 54 secondary metabolites, including 28 hydroxybenzoic, hydroxycinnamic acids, and phenolic glycosides, and 10 triterpene acids, together with 17 flavonoids, were identified or annotated in the assayed *E. spiculifolia* extract using liquid chromatography-high-resolution mass spectrometry. The cytotoxic activity of the extract, alongside gallic, protocatechuic, and oleanolic acids as its constituents, was screened against a panel of malignant human cell lines of different origin (LAMA-84, HL-60, MDA-MB-231, MCF-7, and CASKI). The most prominent antiproliferative effect of the studied extract (with IC_50_ 16.6 μg/mL), matched with the highest tumor selectivity (SI > 120), was observed in the LAMA-84 myeloid cells. These findings were further supported by gallic and oleanolic acid (IC_50_ 6.2 and 1.7 μg/mL, respectively), accounting for a more distinct cytotoxicity. The strongest selective antineoplastic activity was achieved towards the triple-negative breast carcinoma cell line MDA-MB-231, with an IC_50_ of 32.5 μg/mL. This study provided compelling evidence for a wide spectrum of *E. spiculifolia* antitumor activity, indicating its potential as a natural alternative for future therapeutic applications.

## 1. Introduction

*Erica spiculifolia* Salisb. (formerly *Bruckenthalia spiculifolia* (Salisb.) Reichb.) (Ericaceae family) is distributed in Eastern Europe and Western Asia [1]. It is an evergreen shrub growing in the mountain shrublands from 1400 to 2500 m [2]. *E. spiculifolia* is commonly referred to as Balkan heath. As far as we know, there are scarce data on the total flavonoid, tannin, and polyphenol content of the species [3,4]. Thus, the aboveground parts contain 3.71 ± 0.05% flavonoids, 3.80 ± 0.06% tannins, and 4.67 ± 0.08% polyphenols, along with 0.5% hydroquinone derivatives [3]. Dragićević et al. (2024) reported 3.5 ± 0.06 mg rutin equivalents/g flavonoids, 150.17 ± 7.52 mg catechin equivalents (CE)/g tannins, and 194.41 ± 5.74 mg CE/g polyphenols [3,5]. The flavonols quercitrin, isoquercitrin, and quercetin reach 70.91, 32.50, and 3.77 mg/g dry extract, respectively, in the HPLC-UV analysis [5].

A recent study on the phytochemical profiling of *E. spiculifolia* aerial parts revealed the presence of numerous proanthocyanidin oligomers and acylquinic acids in the methanol–aqueous extract, while the chemical markers of the apolar extract were neutral triterpenoids, triterpenoid acids, and phytosterols [6]. The main proanthocyanidins, referred to as hybrid A, B-type oligomers (tri- and tetramers), are characterized by both carbon–carbon and carbon–oxygen interflavan bonds. Among the dominated compounds were chlorogenic acid, (+)-catechin, and quercitrin. The species is considered a rich source of triterpenoid acids such as ursolic and oleanolic acid, reaching up to 32.2 and 6.1 mg/g dw, respectively, together with ursa/olean-2,12-dien-28-oic acids and 3-keto-derivatives. Ursan-type was the most abundant among the triterpenoids, evidenced by the ursolic acid, 3-oxo-ursolic acid, α-amyrin, uvaol, and ursolic aldehyde contents [6]. The essential oil accounted for 0.034% of the aerial part dry mass of *E. spiculifolia* (Bulgarian origin) [7]. α-Terpineol, *endo*-borneol, pinocarveol, and thymol dominated the oxygenated monoterpenes, being present at 7.5, 7.2, 5.9, and 3.7%, respectively. Among the oxygenated sesquiterpenes, caryophyllene oxide, spathulenol, and α-cadinol reached up to 5.0, 2.9, and 2.3%, respectively, together with the sesquiterpene hydrocarbon caryophyllene (4.2%). The ethanol–aqueous extract from Balkan heath aerial parts at 125 μg/mL has been shown to exhibit a notable antioxidant capacity in the β-carotene bleaching assay and against lipid peroxidation, reaching 96% inhibition [3,4,5]. The methanol–aqueous extract from aerial parts scavenged DPPH and ABTS radicals (540.01 and 639.11 mg TE/g), and possessed a high reducing power (660.32 and 869.22 mg TE/g in CUPRAC and FRAP, respectively) and metal chelating capacity (15.57 mg EDTAE/g) [6]. The high antioxidant activity is related to the total phenolic (83.85 mg GAE/g) and flavonoid (78.91 ± 0.41 mg RE/g) content. Caffeoyl conjugates, proanthocyanidin oligomers, catechin alongside α- and β-amyrin, uvaol, and erythrodiol hold significance for their strong antioxidant capacity. As highlighted in the aforementioned study, the beneficial effects of the *E. spiculifolia* extract have also been demonstrated in in vitro enzyme-inhibitory assays, with the evidence suggesting anti-lipase (18.32 mg orlistat equivalents/g), anti-tyrosinase (71.90 mg kojic acid equivalents/g), and anti-α-glucosidase (1.35 mmol acarbose equivalents/g) inhibitory activity. Moderate effects have been evidenced towards acetylcholinesterase α-amylase, elastase, collagenase, and hyaluronidase.

In addition to inducing an antioxidant response and enzyme-inhibitory activity, *E. spiculifolia* extract also displayed anti-inflammatory and immune-modulating properties in both in vitro and in vivo models [4,5,7]. According to recent studies, the reported beneficial effects of Balkan heath leaf, aerial parts, and root extracts could be associated with the arachidonic acid metabolite pathway and the inhibition of NO production [4,7]. Previously, the cytotoxic activity of two pentacyclic triterpenes (ursolic acid and amyrine), isolated from the methanol extract of *Erica andevalensis* aerial parts, has been assessed against three human cancer cell lines, namely TK-10 (renal adenocarcinoma), MCF-7 (breast adenocarcinoma), and UACC-62 (melanoma), and the antimitotic effect in root meristematic cells of *Allium cepa* was also evaluated. Ursolic acid has been reported to exhibit a pronounced cytotoxic activity [8], and multiple studies have recognized its potential as a promising anticancer agent due to its ability to target various cellular pathways involved in tumor progression [9]. Additionally, the cytotoxic potential of infusions obtained from *E. australis* showed antiproliferative activity against hepatocellular carcinoma HepG2 cells, with an IC_50_ value of 278 ± 21 μg/mL [10]. The cytotoxic evaluation of *E. carnea* against RD (human rhabdomyosarcoma), Hep2c (human cervix carcinoma), and L2OB (murine fibroblasts) demonstrated that the subcritical water extract of *E. carnea* exhibited the highest potency [11].

Indeed, extracts of *Erica manipuliflora*, *E. andevalensis*, and *E. glabella* Thunb. demonstrated broad-spectrum cytostatic activity against melanoma, hepatoblastoma, breast, and renal adenocarcinoma cell lines, highlighting the therapeutic potential of *Erica* species [12]. These findings underscore the relevance of Balkan heath as an underexplored source of phytochemicals with promising therapeutic value, particularly due to its prominent content of phenolic compounds, flavonoids, and triterpenoids, which are frequently implicated in antiproliferative and proapoptotic activities [13,14]. A continued investigation into the genus *Erica* may yield valuable insights into the therapeutic properties of its species and help identify novel lead compounds in the development of anticancer agents derived from natural sources.

Despite the accumulating evidence that Balkan heath protects against oxidative stress and inflammation, its cytotoxic activity has not been investigated. In light of prior research, we undertook a comprehensive assessment of the phenolic and triterpene acids, and flavonoid profiles of *E. spiculifolia* methanol–aqueous extract, with a particular focus on evaluating its cytotoxicity and tumor selectivity against a wide variety of human malignancies of diverse origin, including chronic myeloid leukemia (LAMA-84), promyelocytic leukemia (HL-60), triple-negative and hormone-responsive breast carcinoma (MDA-MB-231 and MCF-7, respectively), and cervical cancer (CASKI). The present study was also intended to compare the activity of the Balkan heath extract with that of gallic, protocatechuic, and oleanolic acid as its constituents.

## 2. Results

Based on the MS and MS/MS accurate masses, retention times, fragmentation patterns, relative ion abundance, and comparison with reference standards and the literature data, a total of 54 metabolites were identified or tentatively annotated in *E. spiculifolia* methanol–aqueous extract using ultra-high-performance liquid chromatography coupled with Orbitrap high-resolution mass spectrometry (UHPLC-HRMS) (Table 1). The total ion chromatograms of the studied *E. spiculifolia* extract in negative and positive ion modes are presented in Figures S1 and S2. The compounds identified with reference standards during the present study belong to confidence class 1, while the compounds that were putatively annotated belong to level 2 [15].

### 2.1. UHPLC-HRMS Profiling

#### 2.1.1. Phenolic Acids in *E. spiculifolia* Extract

Overall, 19 hydroxybenzoic acids and their glycosides, and 5 hydroxycinnamic acids and glycosides were identified or annotated in the assayed *E. spiculifolia* extract (Figure S3). The fragmentation patterns of hydroxybenzoic and hydroxycinnamic acids and derivatives are reported elsewhere [16,17].

**Figure 1:**
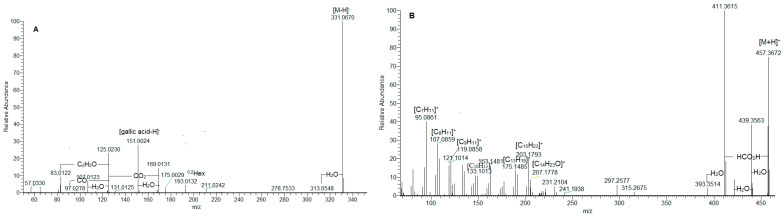

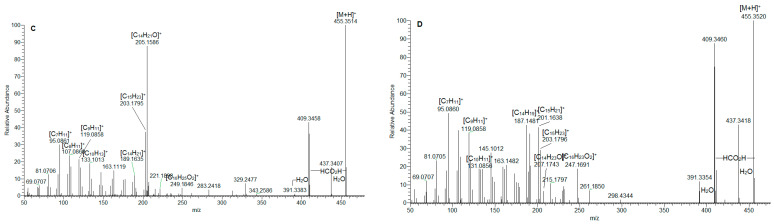
MS/MS spectrum of gallic acid *O*-hexoside (**1**) (**A**); ursolic acid (**36**) (**B**); ursa/olean-dien-28-oic acids (**38**) (**C**); and **33** (**D**).

**Figure 2:**
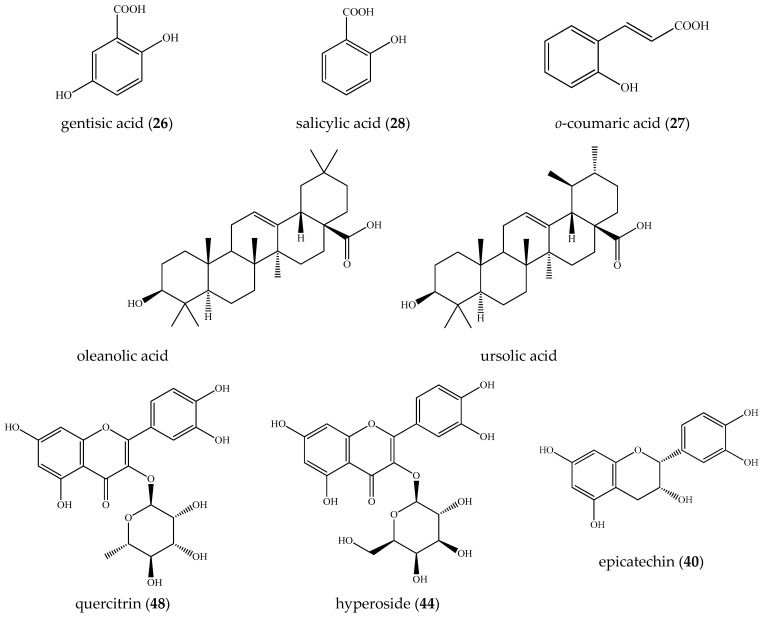
Structures of the main compounds found in *E. spiculifolia*.

Consequently, isobaric **1, 3,** and **8** ([M-H]^−^ at *m*/*z* 331.067), **9**/**21** ([M-H]^−^ at *m*/*z* 299.075), **7**/**10**/**13** ([M-H]^−^ at *m*/*z* 315.073), **14** ([M-H]^−^ at *m*/*z* 329.088), **15** ([M-H]^−^ at *m*/*z* 359.098), **17**/**22** ([M-H]^−^ at *m*/*z* 341.087), and **24** ([M-H]^−^ at *m*/*z* 325.092) were ascribed to gallic acid-, hydroxybenzoic acid-, protocatechuic acid-, vanillic acid-, syringic acid-, caffeic acid-, and coumaric acid-hexosides (Table 1, Figure 1A). Sugar ester **12** afforded indicative fragment ions at *m*/*z* 239.056 [M-H-60]^−^, 209.045 [M-H-90]^−^, and 179.034 [M-H-120]^−^, consistent with the hexose cross ring cleavages ^0,4^Hex, ^0,3^Hex, and ^0,2^Hex, respectively, as has been previously observed [18]. Compound **4**, with [M-H]^−^ at *m*/*z* 271.083 (consistent with C_12_H_15_O_7_) afforded fragment ions at *m*/*z* 151.039 [M-H-C_4_H_8_O_4_]^−^, 109.023 [M-H-C_6_H_10_O_5_]^−^, and 108.020 [M-H-C_6_H_11_O_5_]^−^, suggesting the loss of 120 Da (hexose cross ring, ^0,2^Hex) and 162 Da (hexose residue) (Figure S6). The low abundant fragment ions at *m*/*z* 161.044 [C_6_H_9_O_5_]^−^, 85.028 [Hex-C_2_H_4_O_3_]^−^, 71.012 [Hex-C_3_H_6_O_3_]^−^, and 59.012 [Hex-C_4_H_6_O_3_]^−^ were generated, as has been seen in the reference standard of arbutin (Table 1). In addition, arbutin formic acid adduct (**5**) was also observed. Thus, the mass spectrum at 0.98 min consisted of two ions at *m*/*z* 271.083 and 317.089 [M-H+HCO_2_H]^−^; the former was a deprotonated arbutin, while the latter was a formic acid adduct (consistent with C_13_H_17_O_9_), which confirmed deprotonated molecule [M-H]^−^ [19,20,21]. Compounds **4**, **6**, **11**, **18**, **19**, **20**, **23**, and **25**–**28** were unambiguously identified by a comparison with reference standards (Table 1). Raw MS/MS spectra of the identified compounds in the group are depicted in Figures S6–S15. Among this group, the profile was dominated by gentisic acid (**26**) (6.3%), salicylic acid (**28**) (3.9%), *o*-coumaric acid (**27**) (1.8%), and protocatechuic acid-*O*-hexoside (**10**) (1.1%) (Table 1, Figure 2). Overall, the hydroxybenzoic and hydroxycinnamic acids and phenolic glycosides accounted for 22.4% of the assayed compounds.

#### 2.1.2. Triterpene Acids in *E. spiculifolia* Extract

In (-) ESI-MS, triterpene acids **29**–**32** were annotated in the assayed extract (Table 1). They shared the same [M-H]^−^ at *m*/*z* 487.343, consistent with C_30_H_47_O_5_. The assignment of trihydroxy-urs/olean-en-28-oic acids was based on the fragment ions at *m*/*z* 469.332 [M-H-H_2_O]^−^, 451.322 [M-H-2H_2_O]^−^, 425.342 [M-H-H_2_O-CO_2_]^−^, 411.289 [M-H-2CH_4_-CO_2_]^−^, and 379.300 [M-H-2H_2_O-CO_2_-C_2_H_4_]^−^, suggesting the presence of hydroxyl and carboxyl functional groups. The losses of 18 Da (H_2_O) and 44 Da (CO_2_) have been reported as elimination pathways of hydroxyl and carboxyl functions of triterpenoid acids, respectively, in ESI-MS/MS [22,23]. In (+) ESI-MS, mass spectra of isobaric **36** and **37** at *m*/*z* 457.367 [M+H]^+^, (C_30_H_49_O_3_) were acquired (Table 1, Figure S4). A carboxyl and a hydroxyl group were discernable by the prominent fragment ions at *m*/*z* 439.357 [M+H-H_2_O]^+^, 421.347 [M+H-2H_2_O]^+^, 411.362 [M+H-HCO_2_H]^+^, and 393.352 [(M+H)-H_2_O-HCO_2_H]^+^ [24,25]. The precursor ion yielded RDA ions at *m*/*z* 249.186 [C_16_H_25_O_2_]^+^ (D/E rings) and 207.175 [C_14_H_23_O]^+^ (A/B rings), indicating a Δ^12^ amyrine type with a carboxyl group [26]. The aforementioned assumption was supported by RDA ions at 203.179 [C_15_H_23_]^+^, 189.164 [C_14_H_21_]^+^, 175.149 [C_13_H_19_]^+^, 133.101 [C_10_H_13_]^+^, 119.086 [C_9_H_11_]^+^, 107.086 [C_8_H_11_]^+^, and 95.086 [C_7_H_11_]^+^ (Figure 1B). Thus, **36** and **37** could be referred to as an isobaric pair oleanolic/ursolic acid [25,26,27]. Compound 37 was identified as oleanolic acid through a comparison with reference standards and the literature data [24,25]. Compounds **33**–**35** and **38** shared [M+H]^+^ at *m*/*z* 455.351(2), consistent with C_30_H_47_O_3_. Exemplified by **38**, RDA ions at 249.185 [C_16_H_25_O_2_]^+^ and 205.159 [C_14_H_21_O]^+^ suggested 2 Da less in A/B rings than those in **36**/**37** and an additional double bond (Figure 1C). This assignment was corroborated by the fragment ions at *m*/*z* 203.180 [C_15_H_23_]^+^, 189.164 [C_14_H_21_]^+^, 175.148 [C_13_H_19_]^+^, 133.101 [C_10_H_13_]^+^, 119.086 [C_9_H_11_]^+^, 107086 [C_8_H_11_]^+^, and 95.086 [C_7_H_11_]^+^ (Table 1). On the other hand, compounds **33** and **34** gave a prominent RDA ion at 247.159 [C_16_H_23_O_2_]^+^, supported by 203.180 [C_15_H_23_]^+^, 201.164 [C_15_H_21_]^+^ 187.148 [C_14_H_19_]^+^, 173.132 [C_13_H_17_]^+^, and 131.086 [C_10_H_11_]^+^_,_ suggesting 2 Da less than the D/E rings in **38**. It appears that the additional double bond is in the E-ring, as is indicated by the fragment ions at *m*/*z* 119.0858, 107.0859, and 95.086 (Table 1, Figure 1D). Accordingly, **33**–**35** and **38** were ascribed to ursa/olean-dien-28-oic acids. It is worth noting that triterpene acids reached up to 22.4% of the annotated compounds, with a prevalence of ursa/olean-dien-28-oic acid isomer 1 (**33**) (5.9%), isomer 2 (**34**) (4.7%), and ursolic acid (**36**) (3.7%) (Table 1).

#### 2.1.3. Flavonoids in *E. spiculifolia* Extract

Fragmentation patterns of compounds **39–55** afforded typical ions of flavonol-*O*- and flavone-*O*-glycosides and their aglycones (Table 1, Figure S5) [16,17]. The neutral losses of 146.058, 162.053, 176.032, and 308.111 Da correspond to deoxyhexose, hexose, hexuronic acid, and rutinose, respectively. The annotation of flavonol and flavone aglycones was based on a series of neutral losses of H_2_O (-18 Da), CO (-28 Da), CO_2_ (- 44 Da), and (- CH_2_O (-30 Da). In (-) ESI mode, the precursor ions yielded the prominent ions at *m*/*z* 301.035/300.027 (quercetin along with radical aglycone), 285.040 (luteolin and kaempferol), 269.048 (apigenin), and 315.051/314.043 (isorhamnetin and radical aglycone). Key points in quercetin and kaempferol recognition were the RDA ions at *m*/*z* 178.997 (^1,2^A^−^), 151.003 (^1,3^A^−^), and 107.012 (^0,4^A^−^), together with 135.007 (^0,3^A^−^) (kaempferol) and 121.028 (^1,2^B^−^) (quercetin) (Table 1, Figures S27 and S29). In (+) ESI mode, quercetin and kaempferol were deduced from the RDA ions at *m*/*z* 165.018 (^0,2^A^+^) and 153.019 (^1,3^A^+^), along with (^0,2^B^+^) at *m*/*z* 137.023 and 121.029, respectively (Figures S19 and S20). In (-) ESI mode, luteolin and apigenin yielded prominent RDA ions (^1,3^B^−^) at *m*/*z* 133.028 and 117.033, respectively, corroborated by (^0,2^B^+^) at *m*/*z* 137.023 and 121.029 and (^1,3^B^+^) at *m*/*z* 135.044 and 119.049, respectively (Figures S21, S25, S26 and S28). Compounds **39** and **40** shared the same [M-H]^−^ at *m*/*z* 289.072, consistent with C_15_H_13_O_6_. In (-) ESI mode, precursor ions gave RDA ions at *m*/*z* 137.023 (^1,3^A^−^) and 109.0279 (^1,3^A^−^/CO), supported by *m*/*z* 165.055 (^1,4^B^+^), 139.039 (^1,3^A^+^), and 123.044 (^1,2^B^+^) in (+) ESI mode. Thus, the compounds were assigned as catechin/epicatechin (Figure S17). Based on the comparison with the reference standards, compounds **39**, **41**, **42**, **44**, **46**–**50**, and **52**–**55** were identified as (+) catechin, rutin, isoquercitrin, hyperoside, luteolin 7-*O*-glucoside, quercitrin, isorhamnetin 3-*O*-rutinoside, isorhamnetin 3-*O*-glucoside, apigenin 3-*O*-glucoside, luteolin, quercetin, apigenin, and kaempferol, respectively. Raw MS/MS spectra of the identified flavonoids are depicted in Figures S17–S29. Quercitrin (**48**) (14.0%) was found to be the major compound in the tested extract, followed by epicatechin (**40**) (11.7%) and hyperoside (**44**) (6.9%) (Table 1, Figure 2). Overall, flavonoids were found to be the major group secondary metabolites in the tested extract, accounting for 55.2% of the assayed compounds.

### 2.2. Cytotoxicity Activity

To evaluate the anticancer potential of the studied *E. spiculifolia* extract, we employed a diverse panel of human cancer cell lines alongside a non-malignant control. The hematological models included LAMA-84, a chronic myeloid leukemia line harboring the Philadelphia chromosome [t(9;22)], and HL-60, an acute promyelocytic leukemia line characterized by the t(15;17) translocation. These suspension cultures allowed us to assess the effects on leukemic cells with distinct genetic drivers. In parallel, we investigated the antitumor activity of the plant extract against reproductive system-associated carcinomas, including breast carcinoma cells (MCF-7 and MDA-MB-231) and a cervical cancer cell line (CASKI). MCF-7 cells are hormone-responsive and widely used to model estrogen receptor-positive disease, whereas MDA-MB-231 cells are triple-negative, highly aggressive, and resistant to conventional anti-hormonal therapy. To further broaden the spectrum, we included CASKI, a cervical carcinoma line associated with HPV16 infection, thereby extending the analysis to virally driven epithelial cancer. In addition, normal murine fibroblast cells (CCL-1) were used as a non-malignant comparator, enabling an assessment of selectivity between normal and malignant cells. The presence and degree of tumor selectivity was assessed by calculating the correspondent selectivity indices (SI, selectivity index), defined as the ratio between the half-inhibitory concentrations of the extract in healthy and malignant cells (Table 2).

As presented in Table 2, the *E. spiculifolia* extract demonstrated no detectable cytotoxic activity against normal CCL-1 fibroblasts, even at the highest tested concentrations (>2000 µg/mL), indicating a favorable safety profile. In contrast, all malignant cell lines exhibited significantly lower IC_50_ values compared to CCL-1 (*p* < 0.001), confirming the statistical significance of the extract’s tumor-selective cytotoxicity.

The most notable response was observed in the LAMA-84 myeloid leukemia cell line, which is characterized by the presence of the Philadelphia chromosome (bcr-abl^+^ genotype). In this cell line, the extract exhibited the highest potency (IC_50_ = 16.6 µg/mL) and the most favorable selectivity index (SI = 120.5), implying a highly selective and potentially targeted antiproliferative effect. Notably, this SI value far exceeds those typically reported for well-established chemotherapeutics. For instance, doxorubicin and cisplatin, frequently used as reference drugs in in vitro cytotoxicity assays, generally demonstrate no selectivity or SI values in the range of 1–40, depending on the cancer versus normal cell models applied [28,29,30]. By contrast, the *E. spiculifolia* extract achieved an SI of 120.5 against LAMA-84 cells, suggesting the possibility of a genotype-specific mechanism by some of its constituents, potentially targeting the aberrant BCR-ABL kinase signaling. The referent drug cisplatin, when evaluated against the same leukemic model LAMA-84, demonstrates only moderate cytotoxic efficacy (IC_50_ = 37.8 µM, 11.4 µg/mL) while failing to produce any tumor selectivity, as evinced by the calculated SI < 1. This further underscores the marked advantage of the extract in preferentially targeting malignant over healthy cells.

Interestingly, this activity correlates with the individual cytotoxic profiles of two constituents of the extract—gallic acid (IC_50_ = 6.2 µg/mL, equivalent to 36.4 μM) and oleanolic acid (IC_50_ = 1.7 µg/mL, equivalent to 3.7 μM)—both of which also showed exclusive activity against LAMA-84 cells while remaining inactive against the reproductive cancer cell lines (MDA-MB-231, MCF-7, and CASKI). The selective activity of these compounds toward the bcr-abl^+^ leukemia phenotype raises the possibility of a mechanism involving the inhibition of aberrant kinase signaling pathways, although this hypothesis warrants further mechanistic investigation.

In comparison, the promyelocytic leukemia cell line HL-60, which lacks the *bcr-abl* translocation, demonstrated a markedly reduced but still significant response to the extract, with an IC_50_ of 105.0 µg/mL but a still SI of 19. This difference in sensitivity between the two leukemic models is statistically significant (*p* < 0.001, one-way ANOVA) and underscores the potential genotype-dependent selectivity of the extract’s cytotoxic action. Despite its pronounced cytotoxicity against HL-60 cells (IC_50_ = 8.9 µM, 2.7 µg/mL), the poor selectivity index observed for cisplatin (SI ~ 2.1) indicates a lack of targeted action, as it compromises both malignant and healthy cell viability, in high contrast to the tumor-selective profile of the extract.

Among the carcinoma cell lines, heterogeneity in sensitivity was also evident. The MDA-MB-231 triple-negative breast cancer (TNBC) model showed the highest responsiveness (IC_50_ = 32.5 µg/mL; SI = 61.5), indicating a strong and selective cytotoxic effect. This observation is of particular interest given the aggressive and treatment-resistant nature of TNBC. In contrast, the hormone receptor-positive MCF-7 breast cancer cells exhibited a significantly lower susceptibility (*p* < 0.001), with IC_50_ = 130.1 µg/mL and SI of 15.4, suggesting that the extract may be more effective in hormone-independent breast cancer phenotypes. Notably, with regard to both MDA-MB-231 (IC_50_ = 57.1 µM; SI < 1) and MCF-7 (IC_50_ = 51.6 µM, SI < 1), the reference drug cisplatin exhibited a modest cytotoxic potential and IC_50_ values of 57.1 µM (17.2 µg/mL) and 51.6 µM (15.5 µg/mL), respectively, and an inverse selectivity of less than 1-fold (SI < 1), reflecting its inability to distinctly spare non-malignant cells from cytotoxic insult.

Despite their documented activity in breast and other epithelial cancers, oleanolic and gallic acid, at the tested concentrations, did not exert cytotoxic effects on MCF-7 or MDA-MB-231 cells in our study, suggesting they are not the primary drivers of activity in these models. Instead, the observed cytotoxicity, particularly against the triple-negative MDA-MB-231 breast cancer model, may be attributed to other bioactive compounds within the extract, such as isomeric pentacyclic triterpenoids (ursolic acid) or phenolic compounds.

In the context of the identified/annotated phenolic and triterpene acids, much of the research focus has been centered on the prominent cytotoxic effects of triterpene oleanolic and ursolic acids. Given the extract’s rich composition, phenolic acids such as *o*-coumaric acid, as well as other organic acids, such as quinic acid, both annotated in this study, are likely contributors to the antiproliferative effects against MDA-MB-231 and MCF-7 cells, based on their documented ability to modulate apoptotic pathways, mitochondrial function, and stress responses in breast and colorectal cancer models [31,32,33,34,35]. Their synergistic interactions with flavonoids (also highly abundant in the extract) may further enhance cytotoxicity via multi-targeted mechanisms.

As highlighted in recent research studies, the cytotoxic activities of oleanolic and ursolic acid have been demonstrated towards human cancer cell lines, with evidence strongly suggesting that these acids markedly reduced the viability of cancer cell lines and elevate caspase-3 and caspase-8 activity in the low micromolar range [36,37]. At concentrations of 4 and 8 μmol/L, both triterpene acids activated apoptosis in liver cancer cell lines HepG2, Hep3B, HUH7, and HA22T via increasing DNA fragmentation, decreasing mitochondrial membrane potential, and lowering Na^+^/K^+^ -ATPase activity. Consequently, the disruption of the mitochondrial membrane potential leads to an increased membrane permeability and triggers the release of proapoptotic cytochrome c, along with mitochondria-derived caspase activators [36,37]. Oleanolic and ursolic acids displayed cytotoxic effects against hepatocellular carcinoma HuH7 cells, with IC_50_ 100 and 75 μM, respectively [37]. These acids induce apoptosis in hepatocellular HuH7 cells via the intrinsic mitochondria-mediated pathway and downregulation of the X-linked inhibitor of apoptotic protein (XIAP). In contrast to previous studies, which have reported an IC_50_ value of 38.8 μg/mL for oleanolic acid in triple-negative MDA-MB-231 breast cancer cells 231 [38], our results show an IC_50_ exceeding 500 μg/mL, indicating a substantially lower cytotoxic potency under our experimental conditions against this particular tumor model.

Significant advances have been made in understanding the mechanisms underlying the anti-invasive and anti-metastasis effects of oleanolic and ursolic acids in cancer cells [36]. These acids suppressed both cancer cell adhesion and angiogenesis via attenuating the cell adhesion molecule ICAM-1 production and lowering the angiogenic vascular endothelial growth factor (VEGF) responsible for angiogenesis. In addition to evoking cytotoxic effects on non-small cell lung cancer cell lines A459 and H460 by increasing the expression of proapoptotic protein Bax and altering the Bcl-2/Bax balance, oleanolic acid lowered the expression of the surviving anti-apoptotic protein [39]. It is worth noting that the oleanolic acid decreased the development of melanoma-induced lung metastasis by lowering the aforementioned factor VEGF.

Among the molecular mechanisms of ursolic acid-induced growth inhibition against the breast cancer cell lines MCF-7 and MDA-MB-231 T47) are the downregulation of anti-apoptotic Bcl-2 activity [40], the recruitment of the proapoptotic machinery via caspase-8 and 3, and the cleavage of poly (ADP-ribose) polymerase with equi-inhibitory concentrations ranging be 26 to 53 μM, depending on the exposure time and assay conditions [41,42].

Previous studies have also revealed apoptosis induction through the inhibition of STAT3 and NFK B1 and the activation of mitogen-activated protein kinase (MAPK 8/9/10 and MARK 1/3) in the MCF-7 cell line [43]. Ursolic acid at 25 μM induced an autophagy-mediated endoplasmic reticulum stress [43]. Particularly, MAPK1/3 (not MAPK8/9/10 and MAPK11/12/13/14) appears to be a crucial mediator in ursolic acid-induced autophagy in MCF-7 cells. Luo et al. (2017, 33 ot UA) reported that ursolic acid inhibits cell proliferation and inflammation in MCF-7 and MDA-MB-231 cell lines, and induces autophagy and apoptosis via the glycogen synthase kinase and Bcl-2/caspase-3 signaling pathways [44]. Furthermore, ursolic acid suppresses cell migration and metastasis by the inhibition of c-jun N-terminal kinase (JNK) and by the downregulation of matrix metalloproteinase-2 [45]. Ursolic acid has been found to inhibit the proliferation of a series of colorectal cancer cell lines including HCT15, CO115, HT-29, SW480, HT116, LoVo, and RKO [45]. It displayed cytotoxic activity towards HCT116 and SW480, with IC_50_ values of 13.0 and 10.2 mmol/L. The molecular targets and signaling pathways of ursolic acid involved apoptosis via the activation of the phosphoinositide 3-kinase and MAPK/extracellular signal-regulated kinase signaling pathways [46] and inhibition of the epidermal growth factor regulator and/or MAPK pathway [47]. The upregulation of p53, nuclear factor-jB, Bax, and p21, followed by the activation of caspase-3 and -9 [48], as well as the downregulation of Bcl-2, Bcl-xL, and survivin, have been reported [49].

Moreover, numerous oleanolic acid derivatives have been synthesized, and a marked cytotoxic activity towards KB, MCF-7, HeLa, Hep-G2, 549, KBR-3, SKOV-3, PC-3, and U-87 cancer cell lines has been established, with IC_50_ values in the low micromolar concentration range [50]. Amide and ester derivatives represent the most prevalent semisynthetic modifications of ursolic acid, with several showing an enhanced cytotoxic activity compared to ursolic acid itself against cell lines such as HL-60, HeLa, BGC, and Bel-740 [45].

Despite the relatively long exposure time of 72 h used in this study, gallic acid, protocatechuic acid, and oleanolic acid did not exhibit significant cytotoxicity against MCF-7 or MDA-MB-231 cells at concentrations up to 500 μg/mL. This contrasts with the cited previous reports demonstrating cytotoxic effects of these compounds in various tumor models, including breast cancer malignancies. Such discrepancies may arise from differences in compound purity and formulation, variability in cell line subtypes and culture conditions, as well as distinct assay methodologies and endpoints used across studies. Furthermore, the cellular uptake and metabolism of these compounds can vary depending on experimental settings, potentially influencing the observed activity. Additionally, interactions within the complex extract composition may modulate the bioavailability or efficacy of individual constituents.

Phenolic acids, gallic acid, and their derivatives are among the dominant constituents of the *E. spiculifolia* extract. These compounds have been shown to inhibit the NF-κB-driven expression of antiapoptotic and cell survival factors [51]. In line with these observations, Carcia-Rivera et al. (2011) have reported that gallic acid at 10 μg/mL regulates IκK, IκB kinases, NF-κB, MARK, and MEK1/p90RSK/MSK signaling pathways in MDA-MB-231 breast cancer cells [52]. Accordingly, it lowers the expression of genes of inflammation, metastasis, and anti-apoptosis, such as IL-6/8, COX2, and Bcl-2 [52]. As highlighted in a review article, gallic acid could be a potential anti-inflammatory candidate by inhibiting the NF-κB and MARK signaling pathways [53]. A recent review article emphasizes the anticancer potential of protocatechuic acid and its involvement in the control of various molecular pathways [54]. Protocatechuic acid exerts notable growth inhibiting properties on in vitro models for numerous cancer types, including HepG2 hepatocellular carcinoma, HL-20 leukemia, lung cancer A549, H1299, H3255, colorectal NK, melanoma SK-MEL-28, etc. [55]. Sharma et al. (2019) demonstrated that protocatechuic acid had a dose-dependent cytotoxicity against C6 glioma, MCF-7 breast cancer, and HCT-15 colon cancer cell lines [55]. Overall, this hydroxybenzoic acid interferes with the Bcl-2 family’s activity on anti-apoptotic proteins and caspase-mediated cascade [56]. At a low concentration (1–10 μg/mL), it stimulates the intrinsic apoptosis pathway through the upregulation of p53, Bax, and caspase-9 and activates the extrinsic pathway by modulating caspase-8. Protocatechuic acid can be used in nano-preparations to improve anticancer activity [54].

*p*-coumaric acid also displays cytotoxic activity via the intrinsic apoptosis pathway in a series of colorectal cancer cell lines, including DLD-1, HT-29, SW480, HCT-15, SW-620, and Caco-2 [34]. The treatment with *p*-coumaric acid downregulates Bcl-2 in colorectal carcinoma HCT-15 and HT-29 cells, which is associated with a decrease in mitochondrial membrane potential [34]. Moreover, *p*-coumaric acid downregulates glucose-regulated protein 78 (GRP78) activation in vitro, as assessed on HT-29 and SW480 cells [55]. Indeed, GRP78 is overexpressed in various cancer cells, leading to an increase in the aggressiveness of the cancer [57]. The polyol quinic acid also displays cytotoxic activity and induces apoptosis in oral carcinoma SCC 4 cells by lowering Bcl-2 and increasing Bax expression [57]. It is worth noting that quinic acid could enhance the antitumor effect of cisplatin by reducing Akt/phosphor-Akt and cy-clin D1 expression.

Overall, the combined evidence highlights the substantial anticancer potential of triterpene, phenolic acids, and their derivatives, annotated as major components of the studied *E. spiculifolia* extract. These natural compounds exert multifaceted effects on cancer cell lines by modulating key signaling pathways, including NF-κB, MAPK, and PI3K/Akt, inducing apoptosis, inhibiting proliferation, and suppressing angiogenesis. The consistent cytotoxic activity across various cancer types underscores their promise as therapeutic candidates. A further exploration of their molecular targets and optimization through structural modifications may enhance their clinical relevance in cancer treatment strategies.

## 3. Materials and Methods

### 3.1. Plant Material

*Erica spiculifolia* aerial parts were collected at the locality “Kamen del”, Vitosha Mt. (1906 m. a.s.l.), Bulgaria, during the full flowering stage in July 2024. The species was identified by a member of our research team (D. Zheleva) according to https://www.worldfloraonline.org/ (accessed on 10 september 2024) [1]. A voucher specimen was deposited at the Herbarium of the Institute of Biodiversity and Ecosystem Research, Bulgarian Academy of Sciences (SOM) (Voucher specimen No. 179374). The plant material was dried at room temperature.

### 3.2. Sample Extraction

*E. spiculifolia* aerial parts were powdered by a grinder (Rohnson, R-942, 220–240 V, 50/60 Hz, 200 W, Prague, Czech Republic). Powdered plant material (50 g) was extracted with 80% MeOH (1:20 *w*/*v*) by sonication (100 kHz, ultra-sound bath Biobase UC-20C) for 15 min (×2) at room temperature. The methanol was evaporated in vacuo (40 °C), and water residues were lyophilized (lyophilizer Biobase BK-FD10P; −65 °C) to yield 5.6 g of crude extract. Then, the lyophilized extracts were dissolved in 80% methanol (0.1 mg/mL), filtered through a 0.45 μm syringe filter (Polypure II, Alltech, Lokeren, Belgium), and an aliquot (2 mL) of each solution was subjected to LC–HRMS analyses. The same extract was used for cytotoxicity experiments.

### 3.3. Chemicals

Acetonitrile (hypergrade for LC–MS), formic acid (for LC–MS), and methanol (analytical grade) were provided from Chromasolv (Sofia, Bulgaria). The reference standards (arbutin, gallic, 4-hydroxybenzoic, 3-hydroxybenzoic, caffeic, gentisic, *o*-coumaric, *p*-hydroxyphenylacetic, and salicylic acids, (+) catechin, rutin, isoquercitrin, hyperoside, quercitrin, isorhamnetin 3-*O*-glucoside, isorhamnetin 3-*O*-rutinoside, luteolin 7-*O*-glucoside, apigenin 7-*O*-glucoside, luteolin, quercetin, apigenin, kaempferol) used for compound identification were obtained from Extrasynthese (Genay, France). Oleanolic acid was obtained from Sigma-Aldrich (Saint Louis, MO, USA). Cisplatin was provided by Sigma-Aldrich (Burlington, MA, USA).

### 3.4. UHPLC-HRMS

The UHPLC-HRMS analyses were performed as previously described [52] on a Q Exactive Plus mass spectrometer (ThermoFisher Scientific, Inc., Waltham, MA, USA) with a heated electrospray ionization (HESI-II) probe (Thermo-Scientific). The equipment was operated in negative mode within the m/z range of 150 to 1500. The chromatographic separation was achieved on a Kromasil Eter-nityXT C18 (1.8 µm, 2.1 × 100 mm) reversed-phase column, at 40 °C. The LC analyses were run with a mobile phase consisting of 0.1% formic acid (A) and 0.1% formic acid in acetonitrile (B). The run time was 33 min and the flow rate was 0.3 mL/min. The used gradient elution program was as follows: 0–1 min, 0–5% B; 1–20 min, 5–30% B; 20–25 min, 30–50% B; 25–30 min, 50- 70% B; 30–33 min, 70–95%; 33–34 min, 95–5% B. The injection volume was 1 µL, and the flow rate was 300 µL/min. Data were processed by Xcalibur 4.2 (ThermoScientific, Waltham, MA, USA) instrument control/data handling software, and raw files were achieved.

### 3.5. Semi-Quantitative Relative Approach

The obtained raw file was processed by MZmine 2.0 software using a targeted feature detection focusing on precursor ions and corresponding retention times. The parameters, intensity tolerance of 80%, *m*/*z* tolerance of 5 ppm, and t_R_ tolerance of 0.03 min were used to achieve peak area and export the MS1 list. The relative contents are expressed as % peak area of each compound, normalized to the total peak areas of all metabolites.

### 3.6. Cell Lines

The antiproliferative activity of the extract, gallic, oleanolic, and protocatechuic acids was screened against human malignant cell lines of hematological (LAMA-84, HL-60) and epithelial (MDA-MB-231, MCF-7, CASKI) origin, as well as normal murine fibroblast cells (CCL-1). All cell lines were purchased from the German Collection of Microorganisms and Cell Cultures (DSMZ GmbH, Braunschweig, Germany). Cell cultures were cultivated in a growth medium RPMI 1640 supplemented with 10% fetal bovine serum (FBS), 5% L-glutamine, and incubated under standard conditions of 37 °C and 5% humidified CO_2_ atmosphere.

### 3.7. MTT Colorimetric Assay

The cytotoxic potential of the *E. spiculifolia* extract was studied using a validated method for assessing cell viability known as the Mosmann MTT test. The assay is colorimetric and measures the activity of mitochondrial enzymes, reducing the yellow dye MTT (3-(4,5-dimethylthiazol-2-yl)-2,5-diphenyltetrazolium bromide) to violet formazan crystals. According to protocol, exponential-phased cells were harvested and seeded (100 μL/well) in 96-well plates at the appropriate density. Following a 24 h incubation, cells were treated with serial dilutions of the extract in the concentration range 1000–31.3 μg/mL. Following a 72 h exposure, filter sterilized MTT substrate solution (5 mg/mL in PBS) was added to each well of the culture plate. A further 4h incubation allowed the reduction of the yellow MTT reagent into purple formazan crystals in metabolically active viable cells, which were dissolved in an isopropyl alcohol solution containing 5% formic acid prior to absorbance measurement at 550 nm. Untreated cells (100% cell viability) were used as negative control. Cisplatin was used as positive control.

### 3.8. Statistical Methods

Collected absorbance values were blanked against MTT and isopropanol solution and normalized to the mean value of untreated control (100% cell viability). The obtained data were fitted to “concentration-effect” curves and analyzed by means of non-linear regression in the GraphPad Prism 8.0 software. Statistical analyses were performed using one-way analysis of variance (ANOVA) to assess differences among experimental groups. Where significant differences were detected, post hoc comparisons were conducted using Tukey’s test.

## 4. Conclusions

Herein, a total of 54 secondary metabolites, including phenolic and triterpene acids and flavonoids, were dereplicated/annotated by means of ultra-high-performance liquid chromatography and Orbitrap high-resolution mass spectrometry. The percentage ratio of the assayed classes revealed the highest relative content of flavonoids (55.2%), followed by triterpenoid acids (22.4%) and phenolic acids and derivatives (together with malic acid and arbutin) (22.4%). The tested extract revealed cytotoxic activity against all tested malignant human cell lines. The IC_50_ values ranged from 16.6 ± 2.1 μg/mL (LAMA-84) to 320.7 ± 16.2 μg/mL (CASKI). Gallic and oleanolic acid with IC_50_ 6.2 and 1.7 μg/mL, respectively, hold significance for the most pronounced antiproliferative effect of the extract towards LAMA-84. Our findings suggest that the *E. spiculifolia* extract, alongside its constituents gallic and oleanolic acid, possesses a distinct spectrum of antitumor activity, with a marked selectivity toward hematological malignancies bearing the bcr-abl fusion gene and certain aggressive epithelial cancers such as TNBC. The marked difference in response between LAMA-84 cells (carrying the BCR-ABL oncogene) and HL-60 cells (BCR-ABL negative) provides a compelling rationale for further mechanistic investigations into BCR-ABL-mediated signaling pathways as potential cytotoxicity targets of the extract and its components.

The lack of cytotoxicity in normal fibroblasts across all tested samples further supports the potential of the extract as a source of lead compounds with therapeutic relevance and reduced systemic toxicity. Future studies should focus on isolating additional active constituents, elucidating their molecular targets, and evaluating their in vivo efficacy and safety.

## Figures and Tables

**Table 1:** Secondary metabolites in *E. spiculifolia* methanol–aqueous extract and relative content.

№	Identified/Tentatively Annotated Compound	Molecular Formula	Exact Mass [M-H]^−^	Fragmentation Pattern in (-) ESI-MS/MS	t_R_ (min)	Δ ppm	Level of Confidence [15]	Relative Content [%]
**Hydroxybenzoic, hydroxycinnamic acids, and phenolic glycosides**								
1.	gallic acid-hexoside 1	C_13_H_16_O_10_	331.0670	331.0670 (100), 313.0548 (0.4), 211.0242 (2.2), 169.0131 (15.1), 151.0024 (27.9), 125.0230 (24.6), 107.0123 (6.8)	0.73	−0.060	2	0.5
2.	citric/isocitric acid	C_6_H_8_O_7_	191.0197	191.0188 (11.0), 173.0079 (2.0), 154.9975 (0.7), 147.0288 (0.7), 129.0179 (6.5), 111.0072 (100)	0.91	−4.847	2	1.0
3.	gallic acid-hexoside 2	C_13_H_16_O_10_	331.0670	331.0669 (100), 313.0540 (0.4), 211.0241 (1.6), 169.0131 (21.3), 151.0023 (34.1), 125.0230 (28.7), 107.0124 (8.0)	0.97	−0.634	2	1.0
4.	arbutin ^a^	C_12_H_16_O_7_	271.0823	271.0833 (0.9), 151.0388 (6.5), 113.0229 (5.8), 109.0233 (2.2), 108.0202 (100), 85.0279 (8.1), 71.0122 (12.5), 59.0122 (0.5)	0.98	3.630	1	0.05
5.	arbutin-formic acid adduct	C_13_H_18_O_9_	317.0878	317.0887 (1.5), 271.0824 (48.3), 151.0387 (9.0), 113.0228 (9.9), 109.0282 (7.2), 108.0202 (100), 59.0125 (1.8)	0.98	2.664	1	-
6.	gallic acid ^a^	C_7_H_6_O_5_	169.0142	169.0131 (80.4), 151.0024 (100), 125.0229 (22.3), 107.0123 (16.4), 83.0122 (29.1), 65.0016 (13.1)	1.15	−6.665	1	0.04
7.	protocatechuic acid-*O*-hexoside 1	C_13_H_16_O_9_	315.0727	315.0725 (3.3), 153.0180 (100), 125.3009 (0.1), 109.0280 (52.0), 108.0201 (93.1)	1.19	1.031	2	0.3
8.	gallic acid-hexoside 3	C_13_H_16_O_10_	331.0670	331.0670 (100), 313.0563 (5.7), 211.0242 (2.2), 169.0118 (4.5), 168.0052 (29.4), 125.0231 (30.1), 107.0125 (0.5)	1.21	−0.241	2	0.3
9.	hydroxybenzoic acid-*O*-hexoside 1	C_13_H_16_O_8_	299.0778	299.0753 (0.3), 137.0230 (100), 93.0330 (68.5)	1.28	−3.881	2	0.3
10.	protocatechuic acid-*O*-hexoside 2	C_13_H_16_O_9_	315.0727	315.0722 (100), 153.0182 (27.4), 152.0102 (59.6), 123.0076 (2.5), 109.0286 (11.9), 108.0201 (90.8)	1.67	0.047	2	1.1
11.	protocatechuic acid ^a^	C_7_H_6_O_4_	153.0181	153.0181 (19.2), 109.0280 (100), 91.0173 (0.8), 81.0329 (1.5)	2.03	−8.182	1	1.0
12.	hydroxybenzoyl-*O*-hexose	C_13_H_16_O_8_	299.0778	299.0771 (100), 239.0557 (18.4), 209.0446 (5.4), 179.0340 (40.6), 137.0231 (84.3), 119.0336 (13.7), 93.0330 (19.4), 85.0280 (6.9),	2.05	−0.504	2	0.19
13.	protocatechuic acid-*O*-hexoside 3	C_13_H_16_O_9_	315.0727	315.0731 (35.4), 153.0544 (100), 123.0437 (54.6), 109.0280 (25.4)	2.12	2.872	2	0.04
14.	vanillic acid-*O*-hexoside	C_14_H_18_O_9_	329.0875	329.0876 (2.0),167.0338 (100), 152.0102 (21.4), 123.0437 (15.0), 108.0202 (35.0)	2.12	−0.654	2	0.006
15.	syringic acid-*O*-hexoside	C_15_H_20_O_10_	359.0985	359.0982 (9.2), 239.0554 (0.4), 197.0446 (100), 182.0210 (18.3), 166.9973 (7.4), 153.0544 (14.4), 138.0309 (24.6)	2.26	1.671	2	0.09
16.	protocatechuic acid-*O*-pentoside	C_12_H_14_O_8_	285.0616	285.0616 (7.3), 153.0180 (100), 123.0438 (0.7), 109.0279 (48.2)	2.43	0.208	2	0.07
17.	caffeic acid-*O*-hexoside 1	C_15_H_18_O_9_	341.0871	341.0872 (5.0), 179.0339 (100), 135.0437 (58.5), 107.0487 (0.7)	2.42	−1.628	2	0.1
18.	*p*-hydroxyphenylacetic acid ^a^	C_8_H_8_O_3_	151.0401	151.0388 (7.9), 136.0153 (10.1), 123.436 (67.1), 107.0487 (69.1)	2.49	−8.259	1	0.1
19.	4-hydroxybenzoic acid ^a^	C_7_H_6_O_3_	137.0230	137.0230 (100), 119.0124 (1.8), 108.0201 (7.4), 93.0330 (3.4), 65.0379 (0.5)	2.84	−10.052	1	1.0
20.	3-hydroxybenzoic acid ^a^	C_7_H_6_O_3_	137.0230	137.0230 (100), 109.0280 (15.7), 93.0330 (19.4), 65.0379 (0.8)	2.90	−11,585	1	0.9
21.	hydroxybenzoic acid-*O*-hexoside 2	C_13_H_16_O_8_	299.0778	299.0772 (2.1), 137.0230 (100), 93.0330 (48.3), 85.0280 (0.8)	2.99	0.102	2	0.1
22.	caffeic acid-*O*-hexoside 2	C_15_H_18_O_9_	341.0871	341.0875 (32.3), 281.0680 (1.0), 251.0557 (0.6), 221.0453 (0.3), 179.0338 (100), 135.0437 (64.8), 107.0487 (0.4)	3.09	−0.895	2	0.08
23.	quinic acid	C_7_H_12_O_6_	191.0561	191.0551 (100), 173.0443 (1.6), 155.0335 (0.4), 127.0386 (3.6), 111.0436 (1.6), 93.0329 (5.8), 85.0278 (18.3)	3.19	−5.450	2	1.0
24.	coumaric acid-*O*-hexoside	C_15_H_18_O_8_	325.0930	325.0924 (10.8), 163.0388 (100), 145.0282 (6.5), 119.0487 (80.4)	3.35	−1.571	2	1.0
25.	caffeic acid ^a^	C_9_H_8_O_4_	179.0339	179.0337 (22.7), 161.0131 (13.8), 135.0437 (100), 107.0123 (17.5)	3.54	−7.105	1	0.3
26.	gentisic acid ^a^	C_7_H_6_O_4_	153.0180	153.0181 (59.6), 135.0074 (15.9), 125.0233 (0.5), 109.0280 (100), 91.0173 (2.5), 81.0329 (1.3)	3.79	−8.051	1	6.3
27.	*O*-coumaric acid ^a^	C_9_H_8_O_3_	163.0389	163.0388 (9.6), 119.0487 (100), 93.0331 (1.0)	4.55	−7.774	1	1.8
28.	salicylic acid ^a^	C_7_H_6_O_3_	137.0230	137.0230 (20.4), 108.0201 (3.3), 93.0330 (100), 65.0380 (0.5)	6.25	−10.052	1	3.9
**Triterpene acids in (-) ESI and (+) ESI**								
**№**	**Identified/Tentatively Annotated Compound**	**Molecular Formula**	**Exact Mass** **[M-H]^−^/[M+H]^+^**	**Fragmentation Pattern in (-) ESI-MS/MS and** **(+) ESI-MS/MS**	**t_R_** **(min)**	**Δ ppm**	**Level of Confidence** **[15]**	**Relative Content [%]**
29.	trihydroxy-urs/olean-en-28-oic acid 1	C_30_H_48_O_5_	487.3429 [M-H]^−^	(-) ESI: 487.3426 (100), 469.3333 (0.2), 409.3123 (0.2), 397.3134 (0.8) (+) ESI: nd	10.94	−0.570	2	0.02
30.	trihydroxy-urs/olean-en-28-oic acid 2	C_30_H_48_O_5_	487.3429 [M-H]^−^	(-) ESI: 487.3429 (100), 469.3320 (99.8), 425.3422 (1.1), 411.2893 (0.7), 379.3004 (0.7) (+) ESI: nd	13.68	−0.078	2	0.05
31.	trihydroxy-urs/olean-en-28-oic acid 3	C_30_H_48_O_5_	487.3429 [M-H]^−^	(-) ESI: 487.3433 (43.5), 469.3303 (2.2), 453.3372 (100), 451.3215 (9.4), 441.3375 (14.6), 439.3214 (1.6), 423.3302 (2.1), 407.3337 (3.9), 405.3155 (1.9), 389.2850 (1.4) (+) ESI: nd	15.49	0.743	2	0.3
32.	trihydroxy-urs/olean-en-28-oic acid 4	C_30_H_48_O_5_	487.3429 [M-H]^−^	(-) ESI: 487.3427 (100), 469.3315 (2.7), 453.3372 (94.2), 451.3217 (12.1), 441.3378 (8.0), 439.3238 (0.7), 423.3268 (1.6), 407.3325 (8.4) (+) ESI: nd	15.97	−0.385	2	0.3
33.	ursa/olean-dien-28-oic acid 1	C_30_H_46_O_3_	455.3520 [M+H]^+^	(+) ESI: 455.3520 (100), 437.3418 (41.9), 409.4460 (86.3), 411.3617 (18.5), 391.3354 (11.6), 247.1691 (18.1), 207.1743 (6.5), 203.1796 (28.6), 201.1638 (40.4), 187.1481 (44.1), 173.1326 (15.6), 131.0856 (19.0), 119.0858 (39.6), 107.0860 (39.0), 95.0860 (48.0) (-) ESI: nd	20.20	−0.901	2	5.9
34.	ursa/olean-dien-28-oic acid 2	C_30_H_46_O_3_	455.3520 [M+H]^+^	(+) ESI: 455.3513 (100), 437.3411 (40.9), 409.3453 (18.2), 391.3357 (6.9), 247.1688 (21.4), 207.1746 (9.9), 203.1795 (21.6), 201.1637 (19.6), 187.1478 (10.1), 173.1322 (9.2), 131.0855 (8.5), 119.0858 (31.1), 107.0859 (31.1), 95.0861 (29.8) (-) ESI: nd	20.47	−1.366	2	4.7
35.	ursa/olean-dien-28-oic acid 3	C_30_H_46_O_3_	455.3520 [M+H]^+^	(+) ESI: 455.3487 (100), 437.3396 (51.8), 419.3304 (10.9), 409.3445 (2.3), 205.1586 (4.1), 203.1785 (3.5), 189.1637 (14.2), 175.1489 (7.3), 133.1012 (17.1), 119.0858 (22.9), 107.0859 (21.9), 95.0861 (22.6) (-) ESI: nd	21.08	−7.141	2	3.7
36.	ursolic acid	C_30_H_48_O_3_	457.3676 [M+H]^+^	(+) ESI: 457.3672 (87.0), 439.3563 (41.3), 421.3463 (11.3), 411.3615 (100), 393.3514 (3.7), 249.1857 (1.1), 207.1778 (4.4), 189.1635 (13.6), 175.1485 (6.9), 133.1013 (20.2), 119.0858 (18.1), 107.0859 (32.1), 95.0861 (40.9) (-) ESI: nd	21.18	−0.502	2	3.7
37.	oleanolic acid	C_30_H_48_O_3_	457.3676 [M+H]^+^	(+) ESI: 457.3674 (100), 439.3583 (8.3), 421.3470 (4.3), 411.3612 (3.7), 381.3155 (6.3), 203.179 (5.3), 189.164 (5.7), 175.149 (9.7), 133.1011 (13.7), 119.086 (21.7), 95.0861 (40.7) (-) ESI: nd	21.40	−0.485	1	1.2
38.	ursa/olean-dien-28-oic acid 4	C_30_H_46_O_3_	455.3520 [M+H]^+^	(+) ESI: 455.3514 (100), 437.3407 (16.4), 409.3458 (38.8), 391.3383 (2.1), 249.1846 (5.8), 205.1586 (79.6), 203.1795 (28.9), 189.1635 (12.2), 175.1477 (10.0), 133.1014 (17.7), 119.0858 (24.3), 107.0859 (23.1), 95.0861 (38.7) (-) ESI: nd	22.07	−0.835	2	2.6
**Flavonoids in (-) ESI and (+) ESI**								
**№**	**Identified/Tentatively Annotated Compound**	**Molecular Formula**	**Exact Mass** **[M-H]^−^**	**Fragmentation Pattern in (-) ESI-MS/MS and** **(+) ESI-MS/MS**	**t_R_** **(min)**	**Δ ppm**	**Level of Confidence** **[15]**	**Relative Content [%]**
39.	(+) catechin ^a^	C_15_H_14_O_6_	289.0718	(-) ESI: 289.0716 (100), 245.0815 (36.6), 203.0705 (15.7), 179.0340 (9.9), 137.0230 (14.3), 123.0437 (24.5), 109.0279 (33.3) (+) ESI: 291.0859 (15.0), 273.0755 (2.1), 249.0754 (1.5), 231.0653 (0.3), 207.0651 (6.4), 165.0546 (19.9), 147.0440 (15.8), 139.0389 (100), 123.0442 (64.7)	3.12 3.13	−0.161 −1.287	1	6.4
40.	epicatechin	C_15_H_14_O_6_	289.0718	(-) ESI: 289.0716 (100), 245.0815 (36.7), 203.0706 (16.1), 179.0340 (9.6), 137.0230 (13.3), 123.0437 (22.9), 109.0279 (34.9) (+) ESI: 291.0858 (12.0), 273.0751 (1.9), 249.0752 (1.2), 207.0651 (7.2), 165.0546 (17.6), 147.0440 (18.5), 139.0390 (100), 123.0443 (64.6)	3.90 3.89	−0.161 −0.163	2	11.7
41.	rutin ^a^	C_27_H_30_O_16_	609.1464	(-) ESI: 609.1457 (100), 301.0346 (31.6), 300.0273 (55.7), 271.0247 (26.1), 255.0297 (11.2), 243.0291 (5.8), 227.0350 (1.9), 211.0391 (0.5), 199.0394 (0.3), 178.9974 (3.0), 151.0025 (4.6), 107.0123 (1.9) (+) ESI: 611.1579 (2.0), 465.1022 (14.2), 303.0496 (100), 285.0383 (0.7), 257.0442 (2.1), 229.0490 (3.6), 153.0186 (2.7), 137.0233 (3.2), 165.0181 (0.7),	5.09 5.08	−0.686 −4.518	1	2.0
42.	isoquercitrin ^a^	C_21_H_20_O_12_	463.0886	(-) ESI: 463.0880 (100), 301.0345 (36.0), 300.0273 (74.1), 271.0246 (32.8), 255.0295 (14.7), 243.0295 (8.8), 227.0343 (2.2), 178.9976 (2.7), 151.0024 (5.8), 121.0280 (1.2), 107.0123 (2.4) (+) ESI: 465.1028 (1.6), 303.0492 (100), 285.0393 (0.5), 257.0449 (2.1), 229.0486 (3.6), 165.0180 (1.5), 153.0179 (3.7), 137.0230 (2.5)	5.18 5.19	−0.473 0.188	1	6.3
43.	quercetin *O*-hexuronide	C_21_H_18_O_13_	477.0675	(-) ESI: 477.0670 (91.2), 301.0351 (100), 245.0442 (2.6), 178.9978 (12.3), 151.0028 (16.9), 121.0281 (9.1), 107.0123 (4.0) (+) ESI: nd	5.22		2	0.06
44.	hyperoside ^a^	C_21_H_20_O_12_	463.0886	(-) ESI: 463.0880 (100), 301.0346 (45.0), 300.0274 (75.7), 271.0247 (35.3), 255.0295 (16.5), 243.0293 (9.2), 227.0348 (2.9), 178.9976 (2.6), 151.0024 (4.9), 107.0123 (2.8) (+) ESI: 465.1029 (1.3), 303.0496 (100), 285.0387 (0.6), 257.0437 (2.3), 229.0494 (3.7), 165.0181 (1.8), 153.0183 (3.7), 137.0233 (3.6)	5.29 5.29	−0.538 0.403	1	6.9
45.	luteolin *O*-hexuronide	C_21_H_18_O_12_	461.0725	(-) ESI: 461.0724 (61.2), 285.0403 (100), 257.0449 (0.4), 243.0292 (1.2), 217.0502 (1.3), 199.0388 (2.0), 175.0385 (2.8), 151.0024 (5.2), 133.0281 (8.8), 107.0124 (2.0) (+) ESI: 463.0863 (36.8), 287.0545 (100), 269.0443 (0.3), 241.0483 (0.7), 153.0182 (6.8), 135.0441 (2.3), 137.0233 (0.4)	5.39 5.38	−0.215 −1.668	2	0.2
46.	luteolin 7-*O*-glucoside ^a^	C_21_H_20_O_11_	447.0933	(-) ESI: 447.0930 (100), 285.0401 (82.1), 256.0371 (3.1), 227.0340 (1.0), 217.0509 (0.8), 199.0387 (1.8), 151.0025 (4.9), 133.0280 (3.9), 107.0122 (2.8) (+) ESI: 449.1071 (18.5), 287.0545 (100), 269.0444 (0.4), 241.0483 (0.6), 153.0181 (6.0), 137.0232 (0.5), 135.0440 (2.2)	5.39 5.39	−0.592 −1.532	1	0.7
47.	isorhamnetin 3-*O*-ruinoside ^a^	C_28_H_32_O_16_	623.1618	(-) ESI: 623.1607 (100), 315.0507 (76.7), 300.0263 (13.2), 271.0251 (26.5), 255.0293 (10.1), 243.0296 (14.3), 227.0346 (2.2), 199.0388 (4.2), 151.0029 (1.3) (+) ESI: nd	5.81	−1.058	1	0.1
48.	Quercitrin ^a^	C_21_H_20_O_11_	447.0933	(-) ESI: 447.0930 (100), 301.0348 (48.1), 300.0274 (49.3), 271.0246 (24.6), 255.0298 (11.8), 227.0339 (2.0), 178.9974 (3.2), 151.0025 (7.3), 121.0279 (1.9), 107.0123 (2.9) (+) ESI: 449.1075 (2.4), 303.0496 (100), 257.0446 (1.8), 229.0494 (2.8), 165.0181 (1.0), 153.0180 (2.4), 149.0241 (0.6), 137.0233 (2.8)	5.93 5.94	−0.592 −0.797	1	14.0
49.	isorhamnetin 3-*O*-glucoside ^a^	C_22_H_22_O_12_	477.1044	(-) ESI: 477.1036 (100), 315.0505 (9.8), 314.0433 (32.2), 299.0186 (2.0), 271.0245 (16.8), 257.0450 (3.2), 243.0296 (11.8), 199.0398 (2.6), 178.9977 (1.1), 151.0023 (1.3) (+) ESI: nd	6.06	−0.501	1	0.2
50.	apigenin 7-*O*-glucoside ^a^	C_21_H_20_O_10_	431.0983	(-) ESI: 431.0980 (100), 269.0447 (20.1), 240.0422 (3.6), 211.0386 (1.9), 151.0022 (2.3), 117.0331 (0.9), 107.0119 (0.9) (+) ESI: 433.1122 (15.7), 271.0598 (100), 153.0181 (5.3), 121.0288 (0.4), 119.0498 (2.2)	6.09 6.08	−0.951 −1.647	1	0.3
51.	kaempferol *O*-deoxyhexoside	C_21_H_20_O_11_	431.0983	(-) ESI: 431.0979 (100), 285.0400 (79.4), 255.0296 (39.2), 227.0344 (31.3), 211.0394 (2.1), 151.0017 (0.6), 135.0068 (1.0), 107.0120 (1.2) (+) ESI: 433.1137 (0.5), 287.0546 (100), 213.0534 (0.6), 165.0181 (0.6), 153.0178 (3.4), 121.0286 (2.7)	6.61 6.61	1.021 1.817	2	1.8
52.	luteolin ^a^	C_15_H_10_O_6_	285.0405	(-) ESI: 285.0403 (100), 257.0475 (0.1), 241.0505 (0.6), 217.0494 (0.9), 199.0392 (2.1), 175.0389 (3.0), 151.0023 (4.0), 133.0281 (22.7), 107.0124 (4.2) (+) ESI: 287.0546 (100), 269.0441 (0.6), 241.0495 (1.1), 213.0546 (0.2), 179.0337 (0.5), 153.0182 (10.8), 137.0232 (1.1), 135.0441 (4.4)	7.59 7.59	−0.566 −1.444	1	1.4
53.	quercetin ^a^	C_15_H_10_O_7_	301.0354	(-) ESI: 301.0352 (100), 273.0407 (3.0), 257.0454 (1.0), 245.0451 (1.0), 229.0502 (1.0), 211.0383 (0.4), 178.9976 (21.2), 151.0024 (44.2), 121.0280 (13.6), 107.0123 (16.0) (+) ESI: 303.0496 (100), 285.0395 (1.0), 257.0442 (2.5), 229.0494 (5.4), 165.0185 (1.7), 153.0294 (6.9), 137.0234 (5.0)	7.61 7.62	−0.418 −1.086	1	1.9
54.	apigenin ^a^	C_15_H_10_O_5_	269.0457	(-) ESI: 269.0454 (100), 225.0548 (1.7), 201.0550 (1.2), 151.0024 (4.4), 117.0331 (15.2), 107.0123 (5.1) (+) ESI: 271.0597 (100), 253.0483 (0.2), 225.0543 (0.5), 243.0647 (0.7), 163.0392 (0.6), 153.0182 (9.1), 121.0285 (1.3), 119.0493 (4.2)	8.58 8.58	−0.396 −1.623	1	1.0
55.	kaempferol ^a^	C_15_H_9_O_7_	285.0406	(-) ESI: 285.0403 (100), 257.0475 (0.1), 241.0505 (0.6), 217.0494 (0.9), 199.0392 (2.1), 175.0389 (3.0), 151.0023 (4.0), 107.0124 (4.2) (+) ESI: nd	8.84	−0.741	1	0.08

^a^—Compared to reference standard; Level of confidence: 1—compound identified by comparison to the reference standard; 2—putatively annotated compound; nd—not detected.

**Table 2:** In vitro cytotoxicity (IC_50_, μg/mL ± SD) of *E. spiculifolia* extract, (IC_50_, μg/mL ± SD (μM)) for gallic, oleanolic protocatechuic acid, and cisplatin against a panel of malignant human cell lines of different origin and normal murine fibroblast cells.

Cell Line	LAMA-84 ^a^	SI_LAMA-84_	HL-60 ^b^	SI_HL-60_	MDA-MB-231 ^c^	SI_MDA-MB-231_	MCF-7 ^d^	SI_MCF-7_	CASKI ^e^	SI_CASKI_	CCL-1 ^f^
*E. spiculifolia* extract	16.6 ± 2.1	120.5	105.0 ± 8.8	19.0	32.5 ± 4.6	61.5	130.1 ± 12.1	15.4	320.7 ± 16.2	6.2	>2000
gallic acid	6.2 (36.4 μM)	13.7	>500	-	>500	-	>500	-	>500	-	>500
oleanolic acid	1.7 (3.7 μM)	135.1	>500	-	>500	-	>500	-	>500	-	>500
protocatechuic acid	>500 (>3244 μM)	-	>500	-	311	1.6	>500	-	>500	-	>500
cisplatin	11.4 ± 0.99 (37.8 ± 3.3 μM)	<1	2.7 ± 0.4 (8.9 ± 1.4 μM)	2.1	17.2 ± 1.3 (57.1 ± 4.2 μM)	<1	15.5 ± 1.7 (51.6 ± 5.5 μM)	<1	14.6 ± 1.9 (48.6 ± 6.3 μM)	<1	5.6 ± 0.7 (18.7 ± 2.4 μM)

^a^ bcr-abl+ chronic myeloid leukemia; ^b^ promyelocytic leukemia; ^c^ triple-negative breast carcinoma; ^d^ hormone-responsive breast carcinoma; ^e^ cervical cancer; ^f^ normal murine fibroblast cells.

## Data Availability

The original contributions presented in the study are included in the article; further inquiries can be directed to the corresponding author.

## Supplementary Materials

The following supporting information can be downloaded at https://www.mdpi.com/article/10.3390/plants14193063/s1. Figure S1: Total ion chromatogram (TIC) of *E. spiculifolia* extract in negative ion mode; Figure S2: TIC of *E. spiculifolia* extract in positive ion mode; Figure S3: Extracted ion chromatogram of phenolic acids and derivatives in (-) ESI/MS (mass accuracy 5 ppm) (for numbers and fragmentation patterns, see Table 1 in the main document); Figure S4: Extracted ion chromatogram of triterpene acids in (+) ESI/MS (mass accuracy 5 ppm) (for numbers and fragmentation patterns, see Table 1 in the main document); Figure S5: Extracted ion chromatogram of flavonoids in (-) ESI/MS (mass accuracy 5 ppm) (for numbers and fragmentation patterns, see Table 1 in the main document); Figure S6: (-) ESI-MS/MS spectrum of arbutin (4) at *m*/*z* 271.0823 (271.0809–271.0837) (mass accuracy 5 ppm) (for numbers and fragmentation patterns, see Table 1); Figure S7: (-) ESI-MS/MS spectrum of gallic acid (6) at *m*/*z* 169.0142 (169.0134–169.0150) (mass accuracy 5 ppm) (for numbers and fragmentation patterns, see Table 1); Figure S8: (-) ESI-MS/MS spectrum of protocatechuic acid (11) at *m*/*z* 153.0181 (153.0173–153.0189) (mass accuracy 5 ppm) (for numbers and fragmentation patterns, see Table 1); Figure S9: (-) ESI-MS/MS spectrum of *p*-hydroxyphenylacetic acid (18) at *m*/*z* 151.0401 (151.0393–151.0409) (mass accuracy 5 ppm) (for numbers and fragmentation patterns, see Table 1); Figure S10: (-) ESI-MS/MS spectrum of 4-hydroxybenzoic acid (19) at *m*/*z* 137.0230 (137.0223–137.0237) (mass accuracy 5 ppm) (for numbers and fragmentation patterns, see Table 1); Figure S11: (-) ESI-MS/MS spectrum of 3-hydroxybenzoic acid (20) at *m*/*z* 137.0230 (137.0223–137.0237) (mass tolerance 5 ppm) (for numbers and fragmentation patterns, see Table 1); Figure S12: (-) ESI-MS/MS spectrum of caffeic acid (25) at *m*/*z* 179.0339 (179.0330–179.0348) (mass accuracy 5 ppm) (for numbers and fragmentation patterns, see Table 1); Figure S13: (-) ESI-MS/MS spectrum of gentisic acid (26) at *m*/*z* 153.0181 (153.0173–153.0189) (mass accuracy 5 ppm) (for numbers and fragmentation patterns, see Table 1); Figure S14: (-) ESI-MS/MS spectrum of *o*-coumaric acid (27) at *m*/*z* 163.0389 (163.0381–163.0397) (mass accuracy 5 ppm) (for numbers and fragmentation patterns, see Table 1); Figure S15: (-) ESI-MS/MS spectrum of salicylic acid (28) at *m*/*z* 137.0230 (137.0223–137.0237) (mass accuracy 5 ppm) (for numbers and fragmentation patterns, see Table 1); Figure S16: (+) ESI-MS/MS spectrum of oleanolic acid (37) at *m*/*z* 457.3676 (457.3651–457.3697) (mass accuracy 5 ppm) (for numbers and fragmentation patterns, see Table 1); Figure S17: (+) ESI-MS/MS spectrum of (+) catechin (39) at *m*/*z* 291.0863 (291.0848–291.0878) (mass accuracy 5 ppm) (for numbers and fragmentation patterns, see Table 1); Figure S18: (-) ESI-MS/MS spectrum of rutin (41) at *m*/*z* 609.1464 (609.1434–609.1494) (mass accuracy 5 ppm) (for numbers and fragmentation patterns, see Table 1); Figure S19: (+) ESI-MS/MS spectrum of isoquercitrin (42) at *m*/*z* 465.1028 (465.1005–465.1051) (mass accuracy 5 ppm) (for numbers and fragmentation patterns, see Table 1); Figure S20: (+) ESI-MS/MS spectrum of hyperoside (44) at *m*/*z* 465.1028 (465.1005–465.1051) (mass accuracy 5 ppm) (for numbers and fragmentation patterns, see Table 1); Figure S21: (-) ESI-MS/MS spectrum of luteolin 7-*O*-glucoisde (46) at *m*/*z* 447.0933 (447.0911–447.0955) (mass accuracy 5 ppm) (for numbers and fragmentation patterns, see Table 1); Figure S22: (-) ESI-MS/MS spectrum of isorhamnetin 3-*O*-rutinoside (47) at *m*/*z* 623.1618 (623.1587–623.1649) (mass accuracy 5 ppm) (for numbers and fragmentation patterns, see Table 1); Figure S23: (-) ESI-MS/MS spectrum of quercitrin (48) at *m*/*z* 447.0933 (447.0911–447.0955) (mass accuracy 5 ppm) (for numbers and fragmentation patterns, see Table 1); Figure S24: (-) ESI-MS/MS spectrum of isorhamnetin 3-*O*-glucoside (49) at *m*/*z* 477.1044 (477.1020–477.1068) (mass accuracy 5 ppm) (for numbers and fragmentation patterns, see Table 1); Figure S25: (-) ESI-MS/MS spectrum of apigenin 7-*O*-glucoside (50) at *m*/*z* 431.0983 (431.0961–431.1005) (mass accuracy 5 ppm) (for numbers and fragmentation patterns, see Table 1); Figure S26: (-) ESI-MS/MS spectrum of luteolin (52) at *m*/*z* 285.0403 (285.0389–285.0417) (mass accuracy 5 ppm) (for numbers and fragmentation patterns, see Table 1); Figure S27: (-) ESI-MS/MS spectrum of quercetin (53) at *m*/*z* 301.0354 (301.0339–301.0369) (mass accuracy 5 ppm) (for numbers and fragmentation patterns, see Table 1); Figure S28: (-) ESI-MS/MS spectrum of apigenin (54) at *m*/*z* 269.0457 (269.0444–269.0470) (mass accuracy 5 ppm) (for numbers and fragmentation patterns, see Table 1); Figure S29: (-) ESI-MS/MS spectrum of kaempferol (55) at *m*/*z* 285.0403 (285.0389–285.0417) (mass accuracy 5 ppm) (for numbers and fragmentation patterns, see Table 1).
