# Supplementary material for: Insight into Cytotoxic Potential of Erica spiculifolia Salisb (Balkan Heath)
Source: Plants (Basel). 2025 Oct 3;14(19):3063. doi: 10.3390/plants14193063 (PMC12526400; doi:10.3390/plants14193063)
Supplement: Supplementary file 1 [file plants-14-03063-s001.zip › plants-3872120-supplementary.pdf]

Insight into cytotoxic potential of *Erica spiculifolia* Salisb. (Balkan heath)

Reneta Gevrenova [1]*, Rositsa Mihaylova [2], Nikolay Bebrivenski[2], Georgi Momekov [2], and Dimitrina Zheleva-Dimitrova [1]

[1] Department of Pharmacognosy, Faculty of Pharmacy, Medical University of Sofia, 1000 Sofia, Bulgaria; rgevrenova@pharmfac.mu-sofia.bg (R.G.); dzheleva@pharmfac.mu-sofia.bg (D.Z.);
[2] Department of Pharmacology, Pharmacotherapy and Toxicology, Faculty of Pharmacy, Medical University of Sofia, 1000 Sofia, Bulgaria; rmihaylova@pharmfac.mu-sofia.bg (R.M.); nikbebsl@gmail.com (N.B.); gmomekov@pharmfac.mu-sofia.bg (G.M.);

* Correspondence: rgevrenova@pharmfac.mu-sofia.bg (R.G.)

Supplemental material

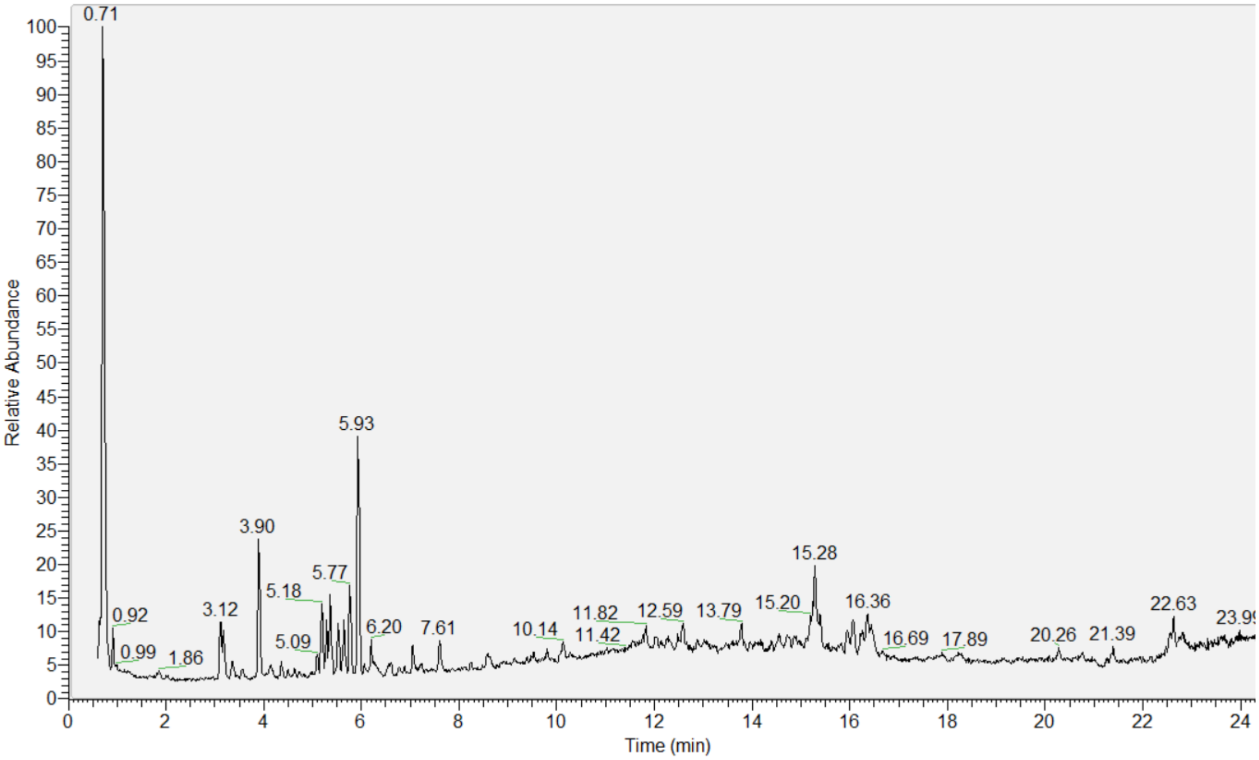

Figure S1. Total ion chromatogram (TIC) of *E. spiculifolia* extract in negative ion mode.

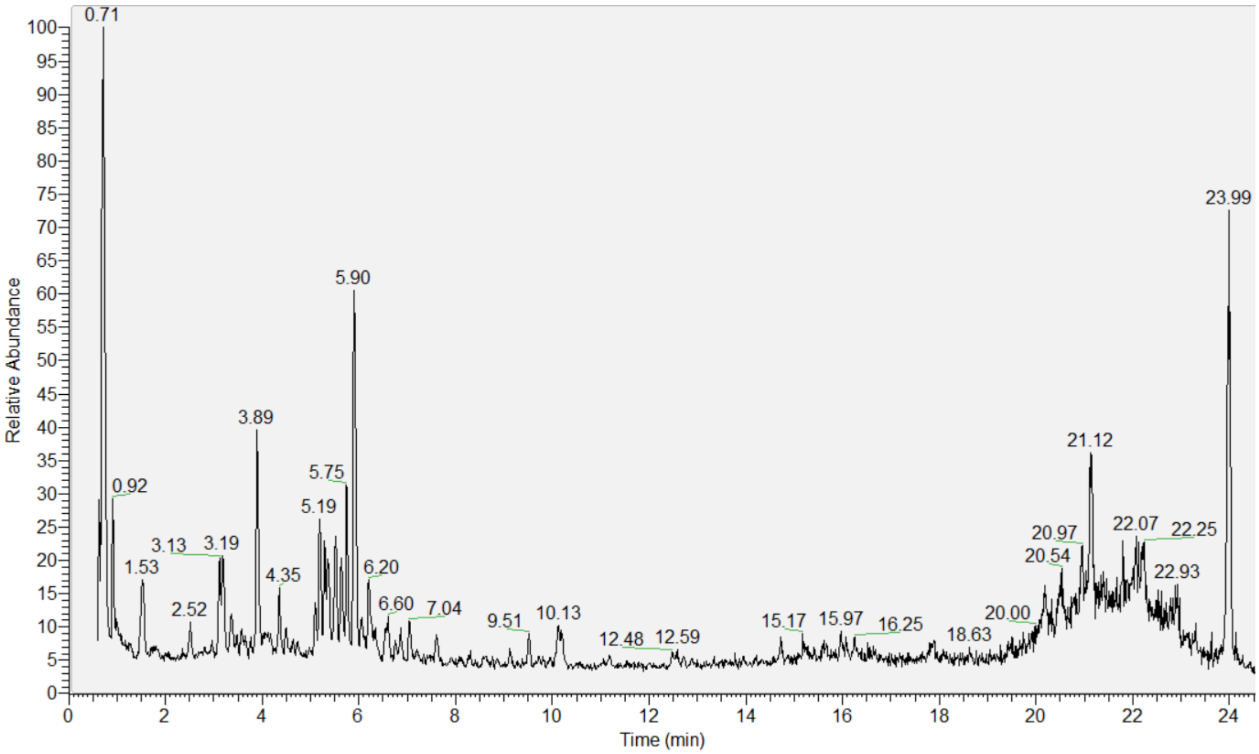

Figure S2. TIC of *E. spiculifolia* extract in positive ion mode.

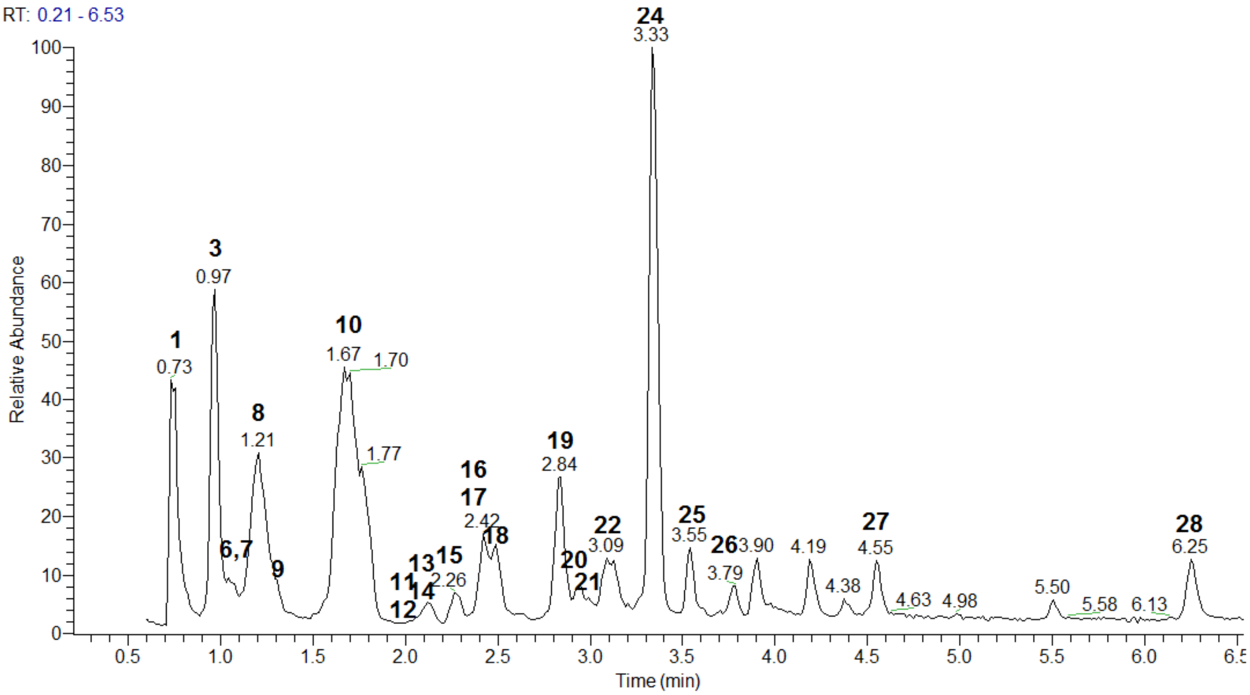

Figure S3. Extracted ion chromatogram of phenolic acids and derivatives in (-) ESI/MS (mass accuracy 5 ppm) (for numbers and fragmentation patterns, see Table 1 in the main document).

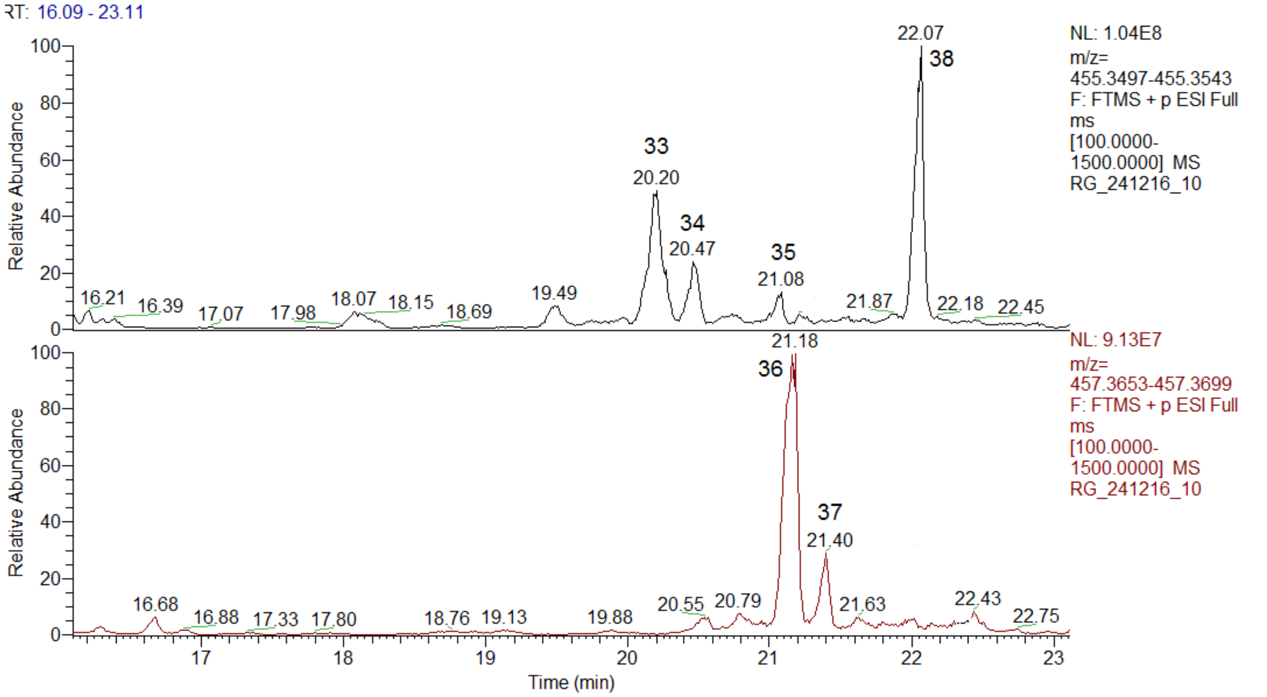

Figure S4. Extracted ion chromatogram of triterpene acids in (+) ESI/MS (mass accuracy 5 ppm) (for numbers and fragmentation patterns, see Table 1 in the main document).

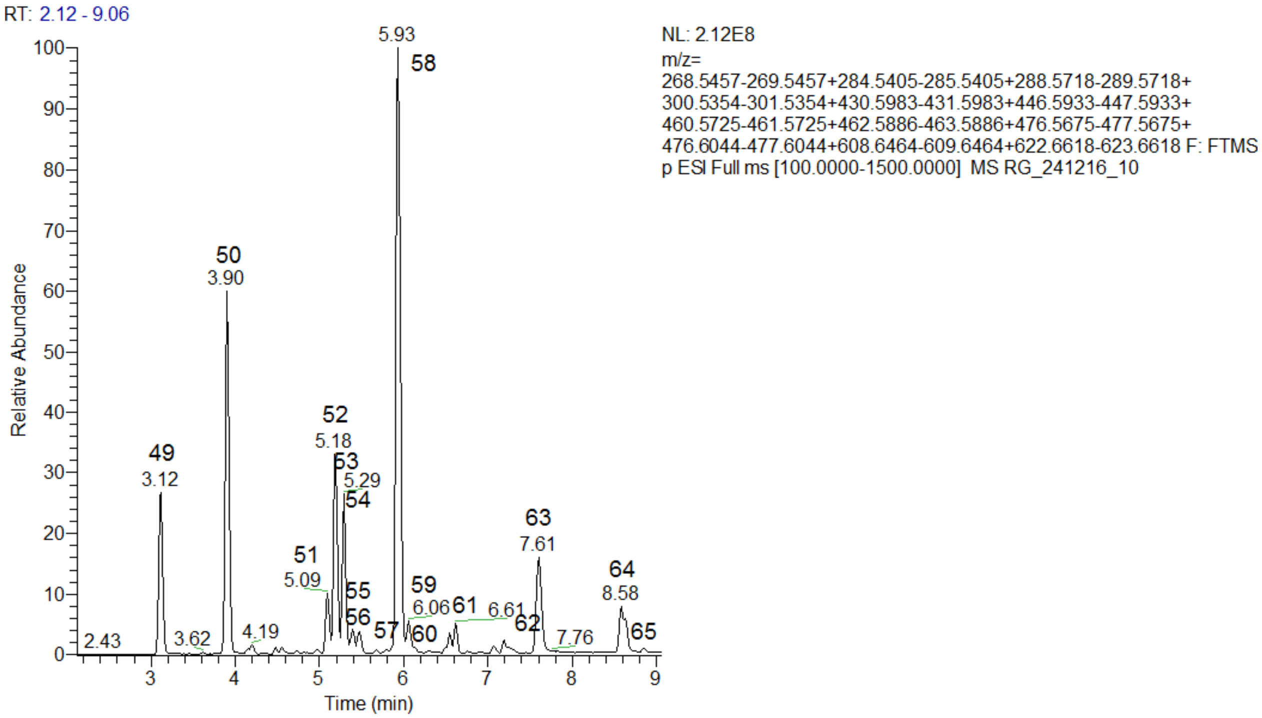

Figure S5. Extracted ion chromatogram of flavonoids in (-) ESI/MS (mass accuracy 5 ppm) (for numbers and fragmentation patterns, see Table 1 in the main document).

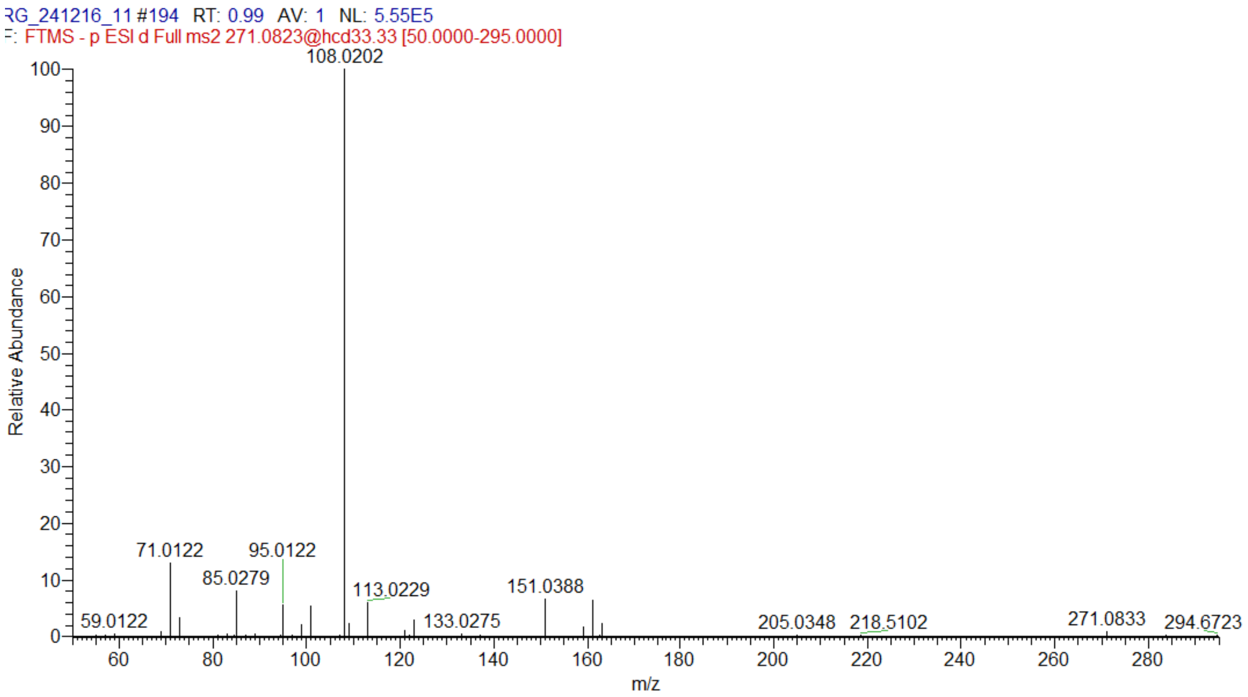

Figure S6. (-) ESI-MS/MS spectrum of arbutin (**4**) at *m/z* 271.0823 (271.0809-271.0837) (mass tolerance 5 ppm) (for numbers and fragmentation patterns, see Table 1).

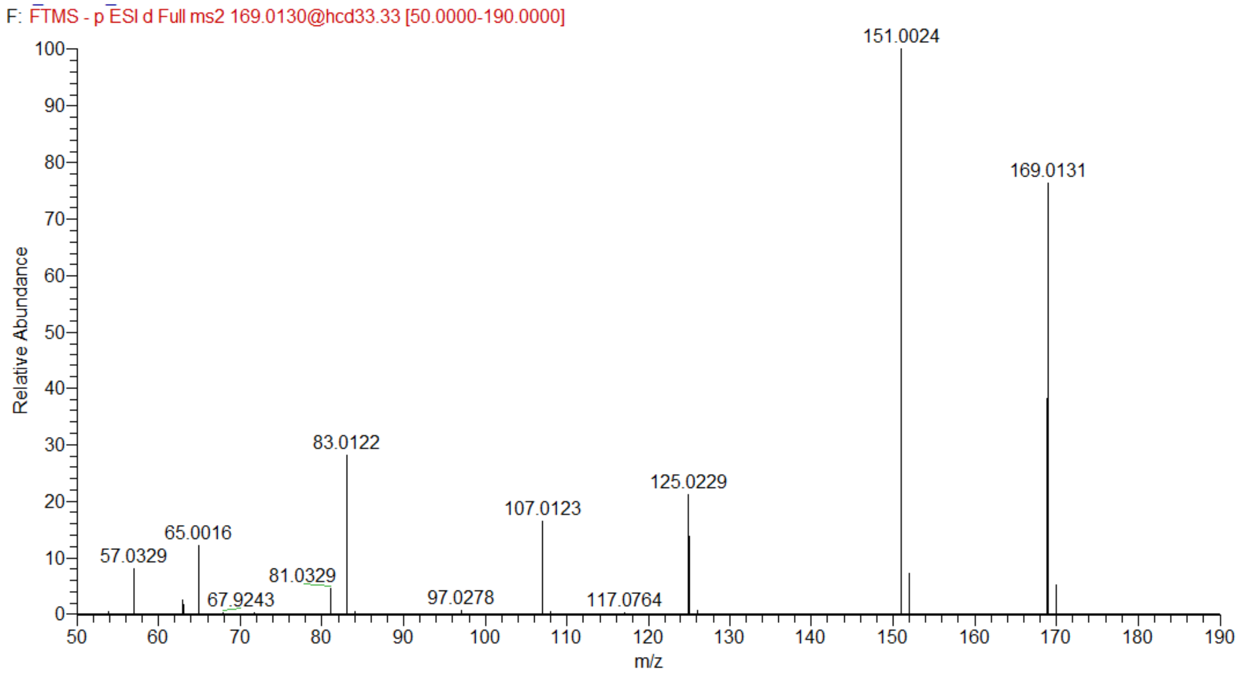

Figure S7. (-) ESI-MS/MS spectrum of gallic acid (**6**) at *m/z* 169.0142 (169.0134-169.0150) (mass tolerance 5 ppm) (for numbers and fragmentation patterns, see Table 1).

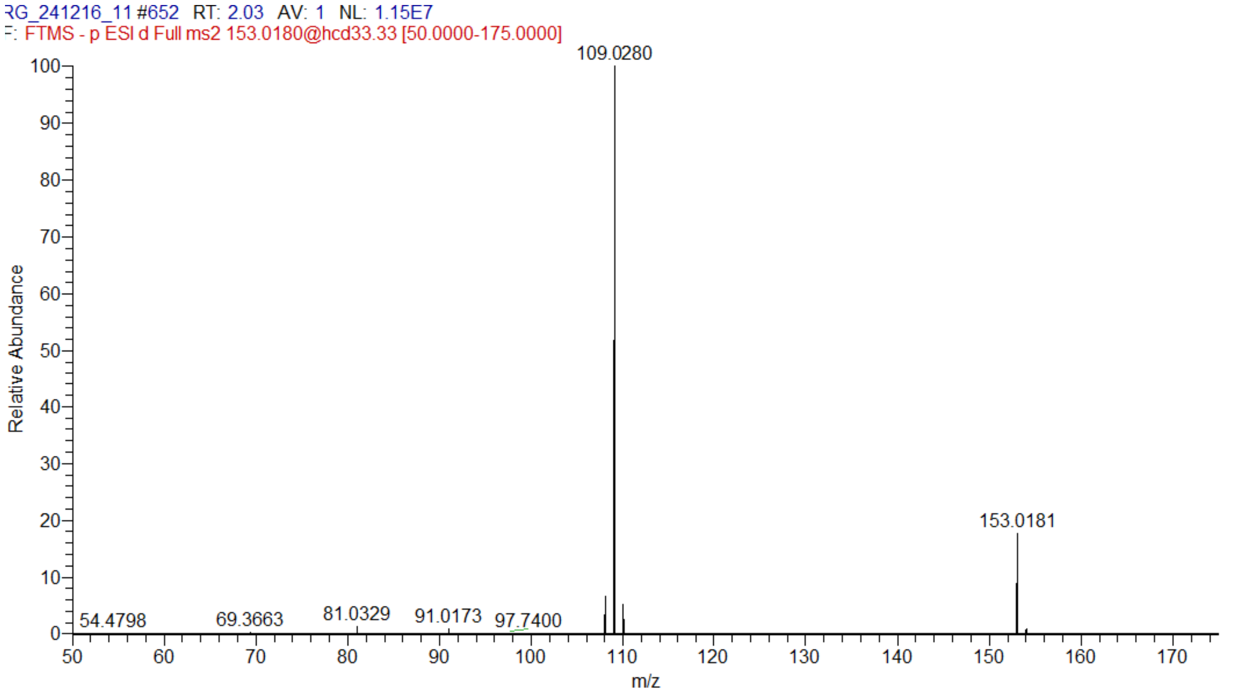

Figure S8. (-) ESI-MS/MS spectrum of protocatechuic acid (**11)** at *m/z* 153.0181 (153.0173-153.0189) (mass tolerance 5 ppm) (for numbers and fragmentation patterns, see Table 1).

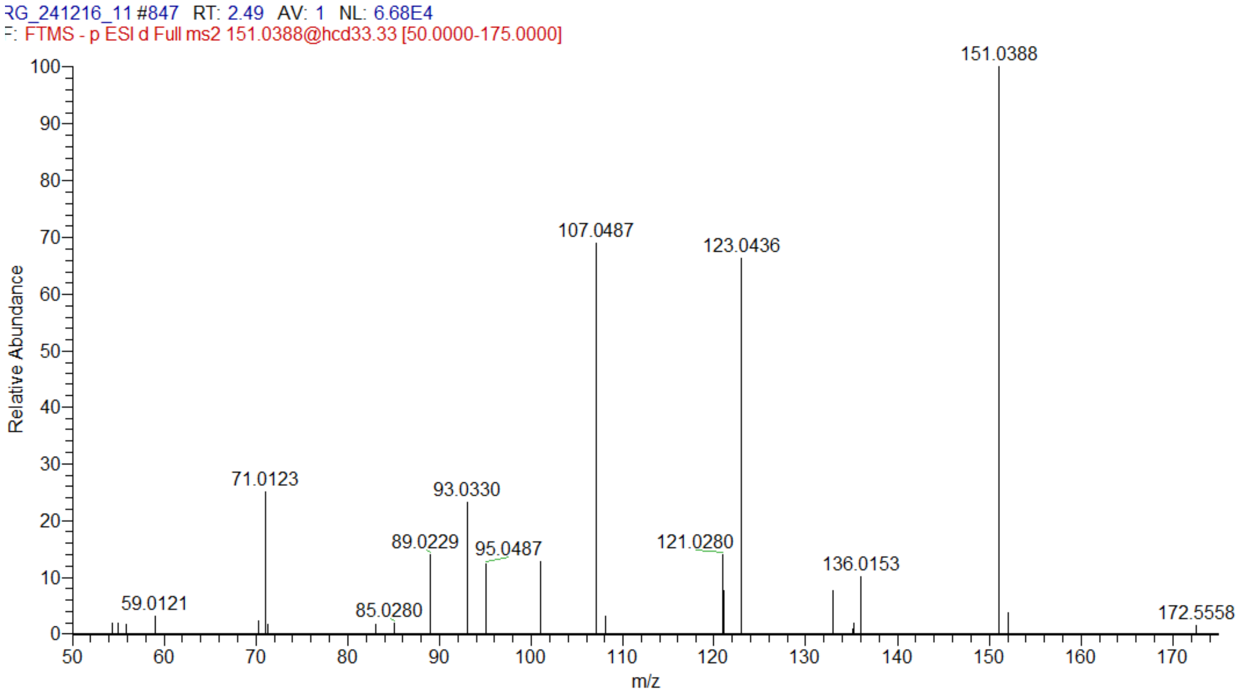

Figure S9. (-) ESI-MS/MS spectrum of *p*-hydroxyphenylacetic acid (**18)** at *m/z* 151.0401 (151.0393-151.0409) (mass tolerance 5 ppm) (for numbers and fragmentation patterns, see Table 1).

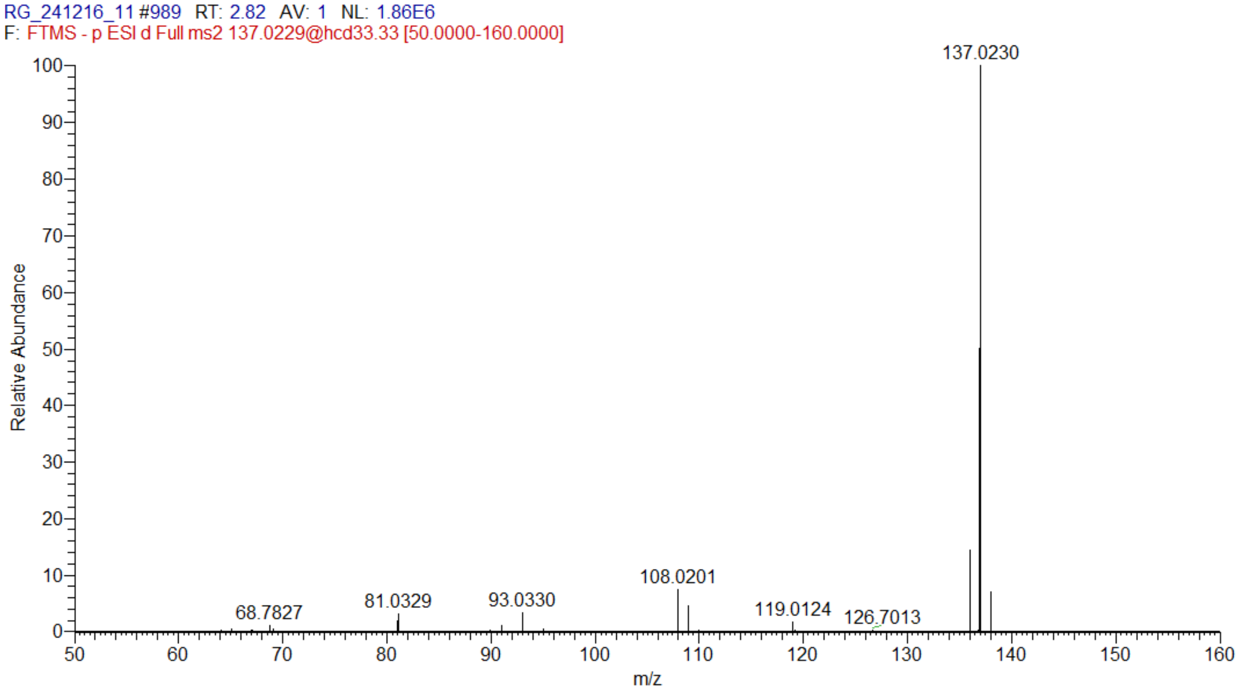

Figure S10. (-) ESI-MS/MS spectrum of 4-hydroxybenzoic acid (**19**) at *m/z* 137.0230 (137.0223-137.0237) (mass tolerance 5 ppm) (for numbers and fragmentation patterns, see Table 1).

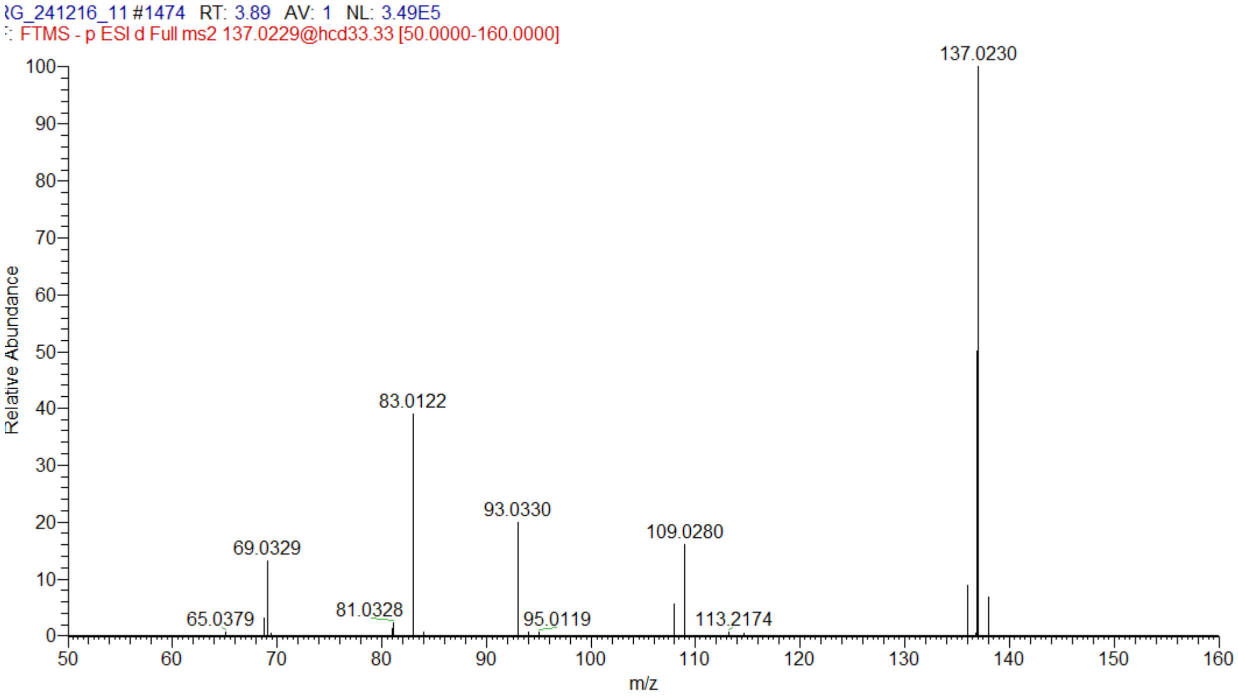

Figure S11. (-) ESI-MS/MS spectrum of 3-hydroxybenzoic acid (**20**) at *m/z* 137.0230 (137.0223-137.0237) (mass tolerance 5 ppm) (for numbers and fragmentation patterns, see Table 1).

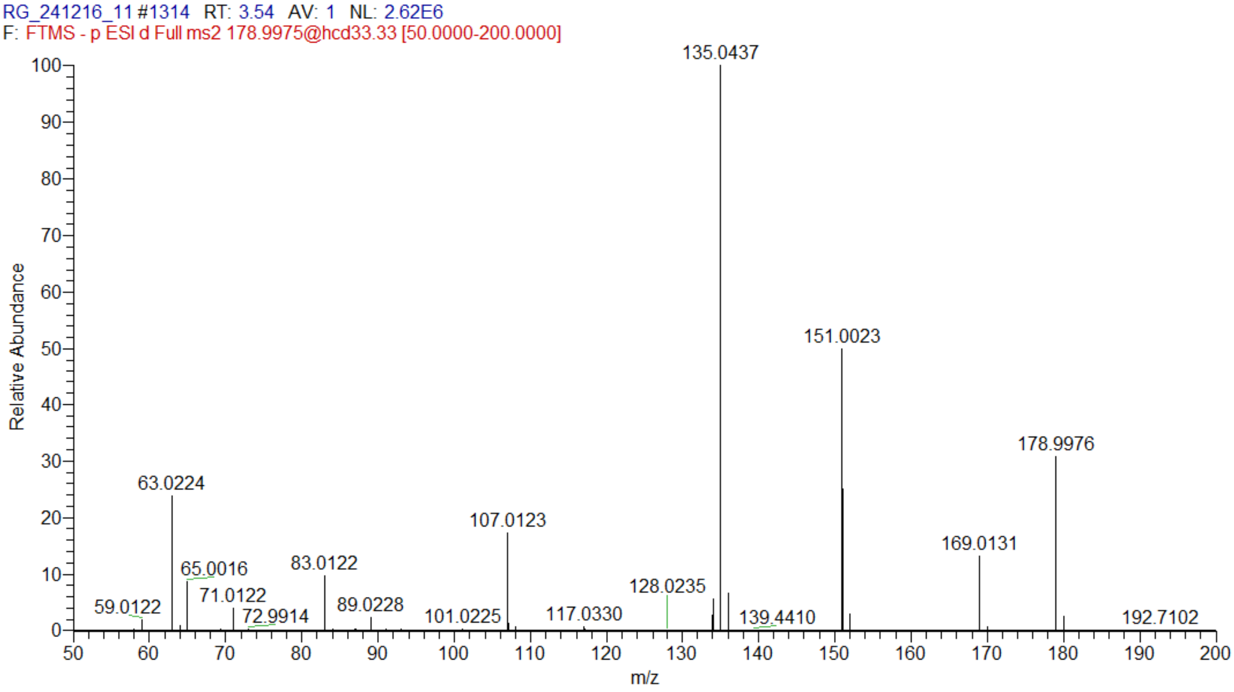

Figure S12. (-) ESI-MS/MS spectrum of caffeic acid (**25**) at *m/z* 179.0339 (179.0330-179.0348) (mass tolerance 5 ppm) (for numbers and fragmentation patterns, see Table 1).

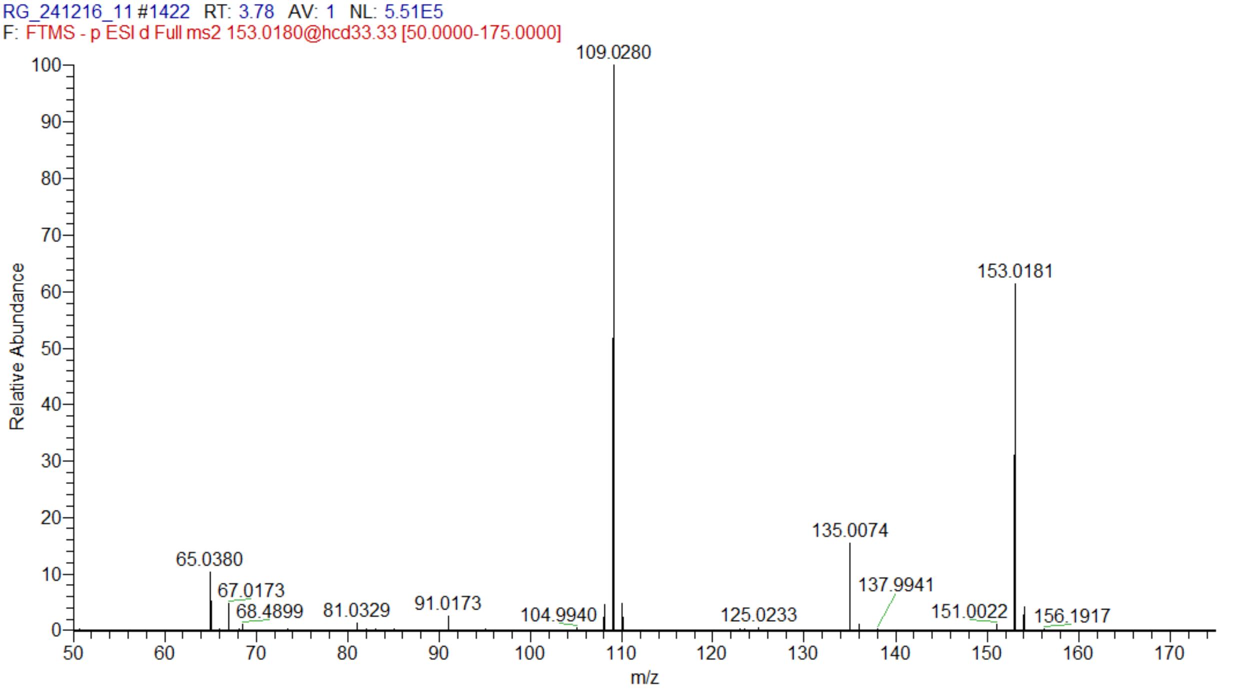

Figure S13. (-) ESI-MS/MS spectrum of gentisic acid (**26**) at *m/z* 153.0181 (153.0173-153.0189) (mass tolerance 5 ppm) (for numbers and fragmentation patterns, see Table 1).

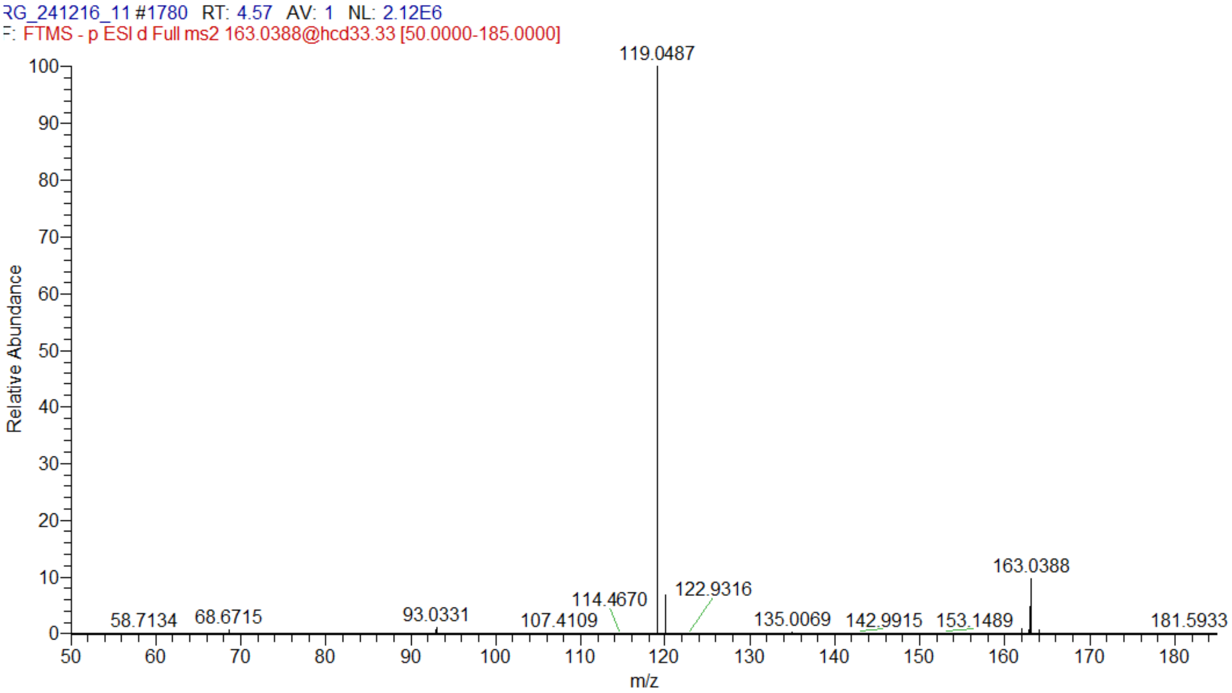

Figure S14. (-) ESI-MS/MS spectrum of *o*-coumaric acid (**27**) at *m/z* 163.0389 (163.0381-163.0397) (mass tolerance 5 ppm) (for numbers and fragmentation patterns, see Table 1).

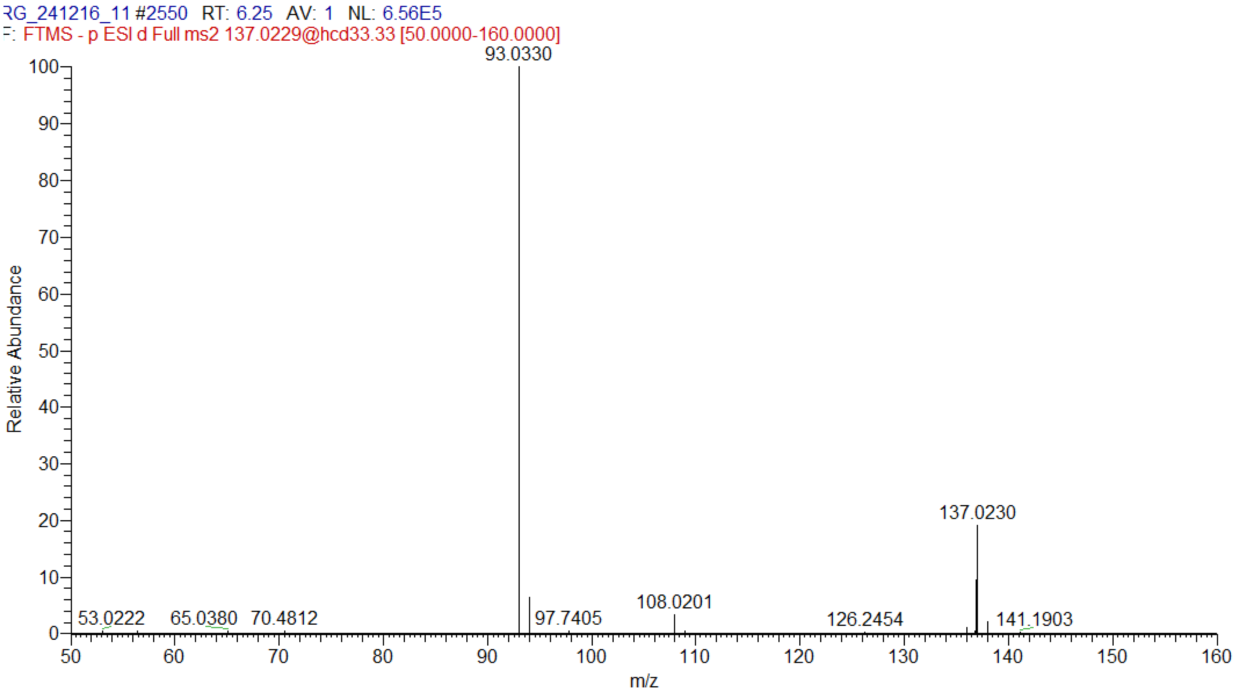

Figure S15. (-) ESI-MS/MS spectrum of salicylic acid (**28**) at *m/z* 137.0230 (137.0223-137.0237) (mass tolerance 5 ppm) (for numbers and fragmentation patterns, see Table 1).

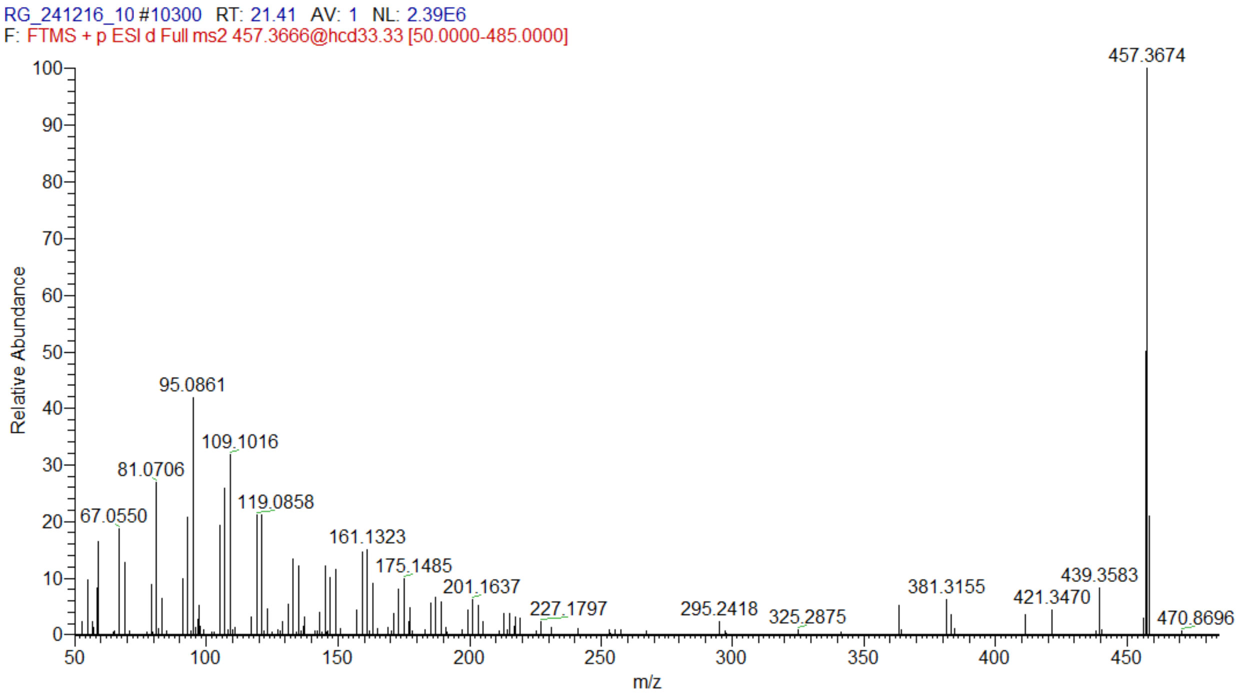

Figure S16. (+) ESI-MS/MS spectrum of oleanolic acid (**37**) at *m/z* 457.3676 (457.3651-457.3697) (mass accuracy 5 ppm) (for numbers and fragmentation patterns, see Table 1).

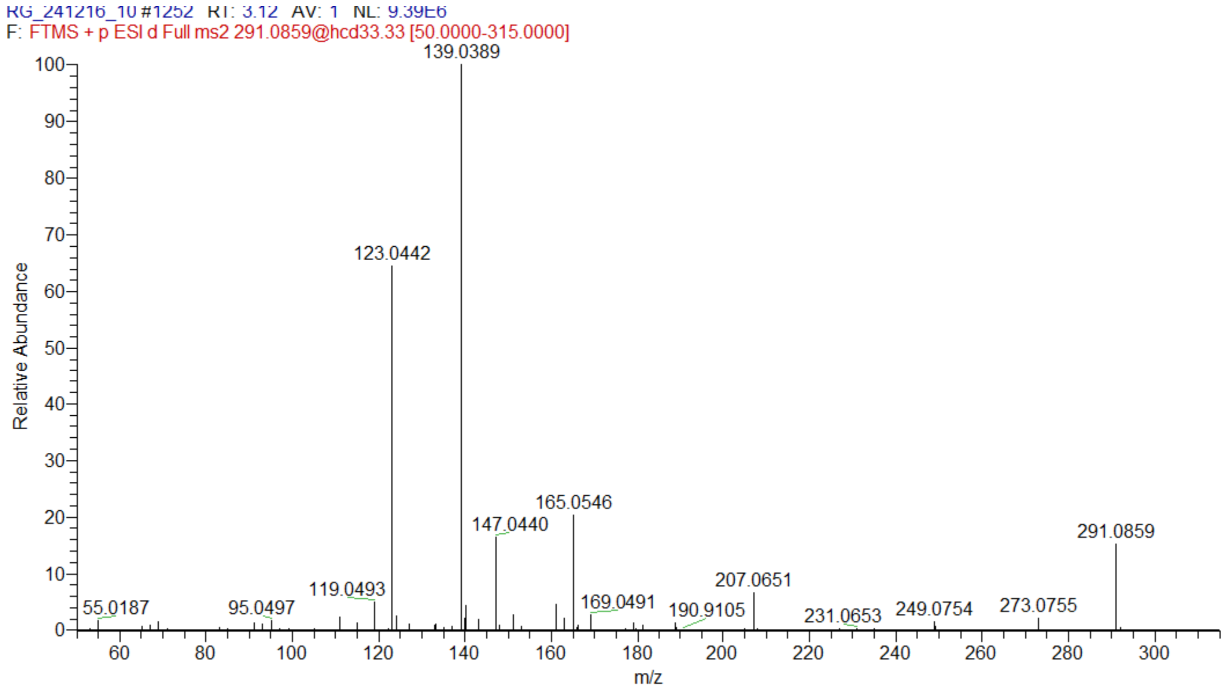

Figure S17. (+) ESI-MS/MS spectrum of (+) catechin (**39**) at *m/z* 291.0863 (291.0848-291.0878) (mass accuracy 5 ppm) (for numbers and fragmentation patterns, see Table 1).

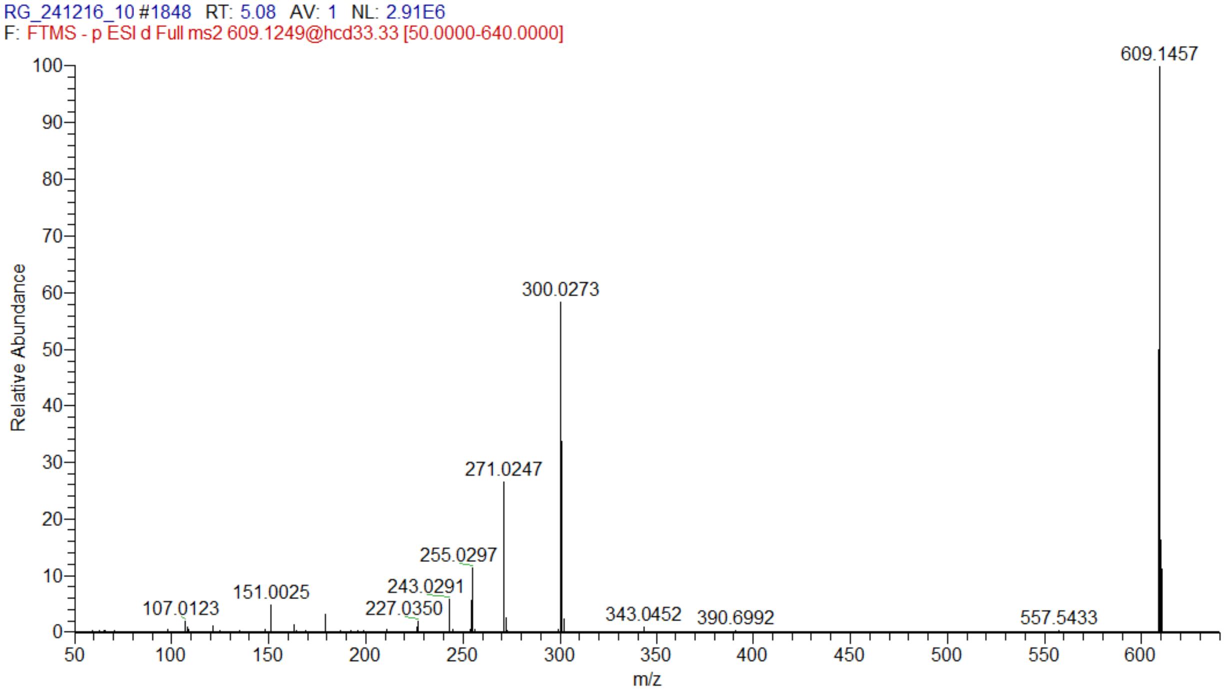

Figure S18. (-) ESI-MS/MS spectrum of rutin (**41)** at *m/z* 609.1464 (609.1434-609.1494) (mass accuracy 5 ppm) (for numbers and fragmentation patterns, see Table 1).

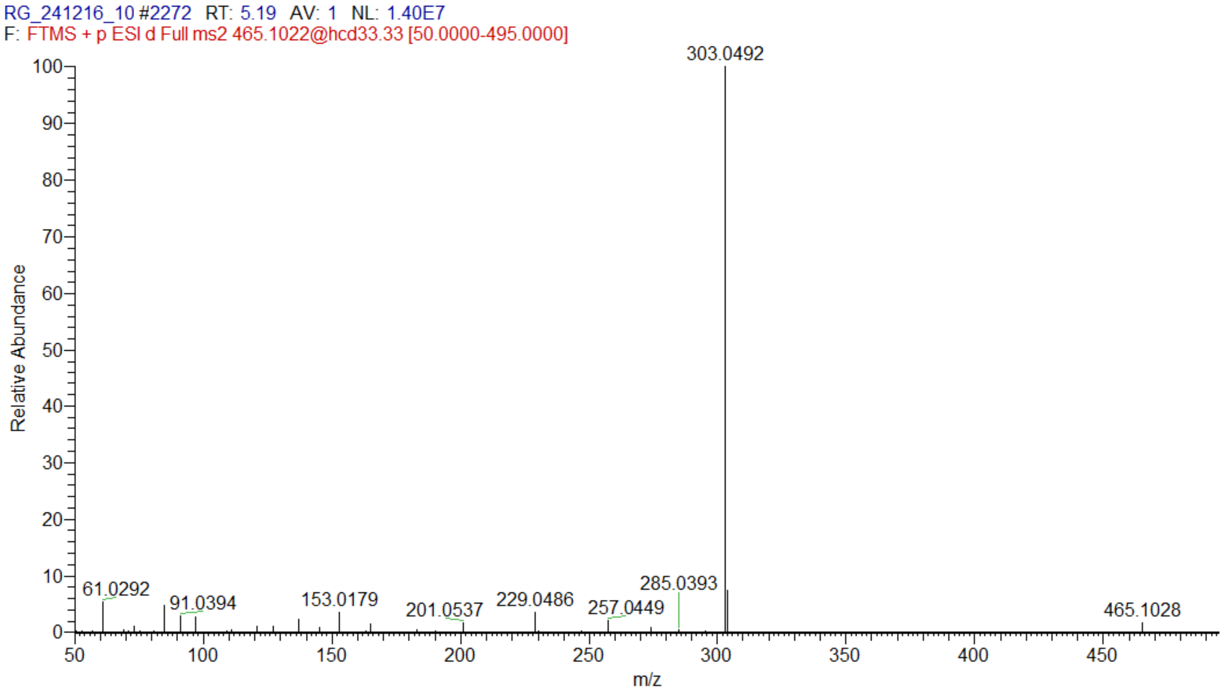

Figure S19. (+) ESI-MS/MS spectrum of isoquercitrin (**42)** at *m/z* 465.1028 (465.1005-465.1051) (mass accuracy 5 ppm) (for numbers and fragmentation patterns, see Table 1).

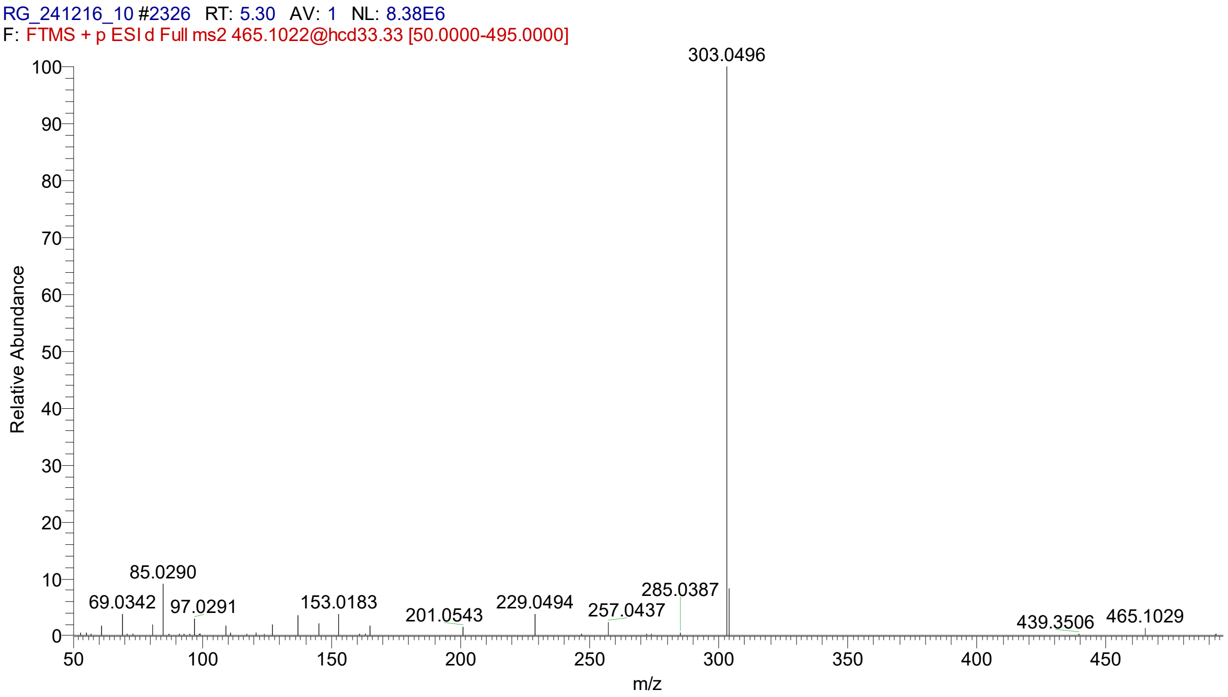

Figure S20. (+) ESI-MS/MS spectrum of hyperoside (**44)** at *m/z* 465.1028 (465.1005-465.1051) (mass accuracy 5 ppm) (for numbers and fragmentation patterns, see Table 1).

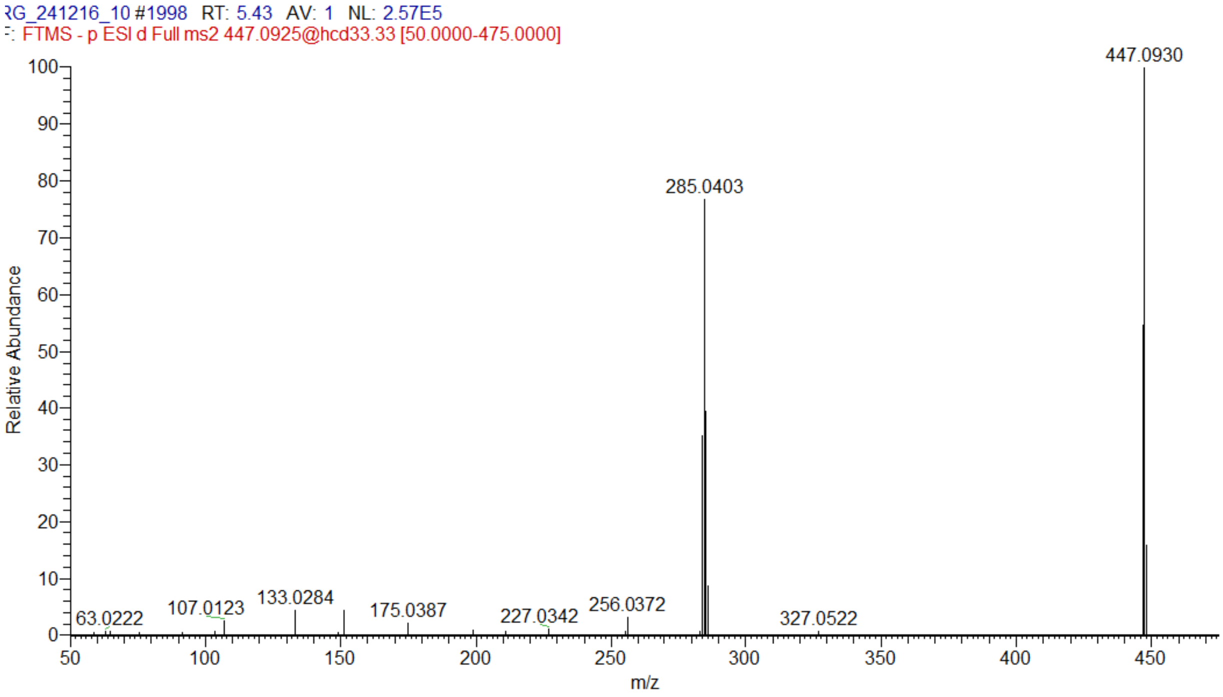

Figure S21. (-) ESI-MS/MS spectrum of luteolin 7-*O*-glucoisde (**46**) at *m/z* 447.0933 (447.0911-447.0955) (mass accuracy 5 ppm) (for numbers and fragmentation patterns, see Table 1).

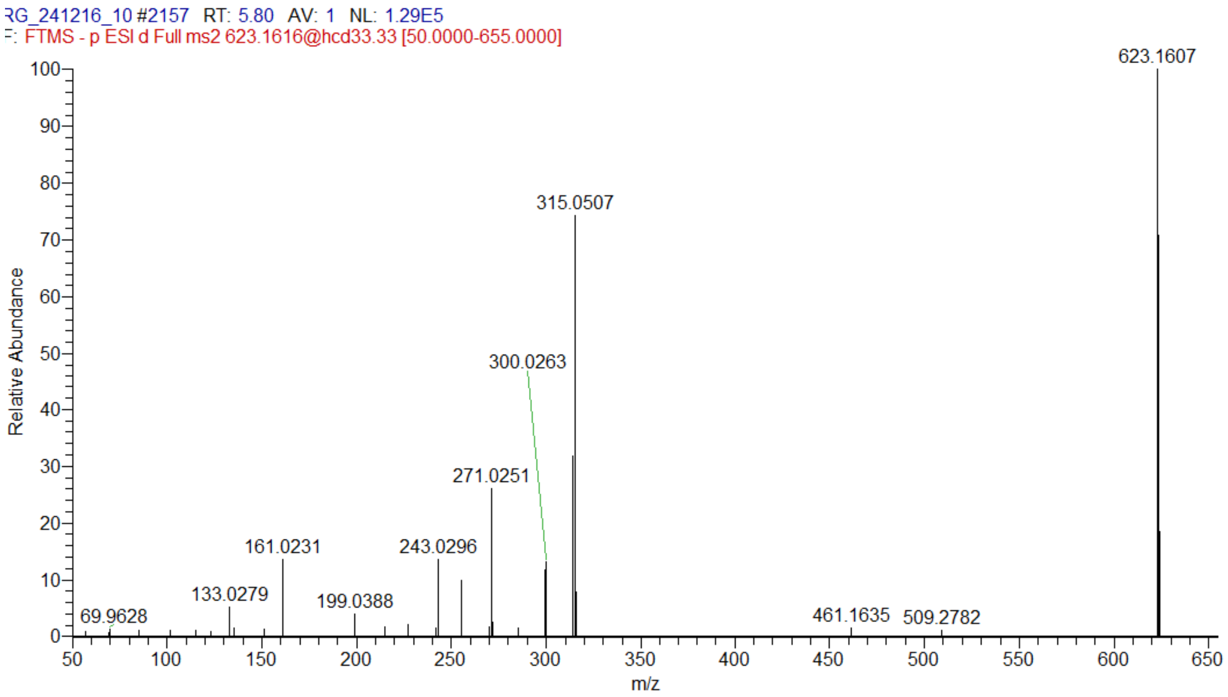

Figure S22. (-) ESI-MS/MS spectrum of isorhamnetin 3-*O*-rutinoside (**47**) at *m/z* 623.1618 (623.1587-623.1649) (mass accuracy 5 ppm) (for numbers and fragmentation patterns, see Table 1).

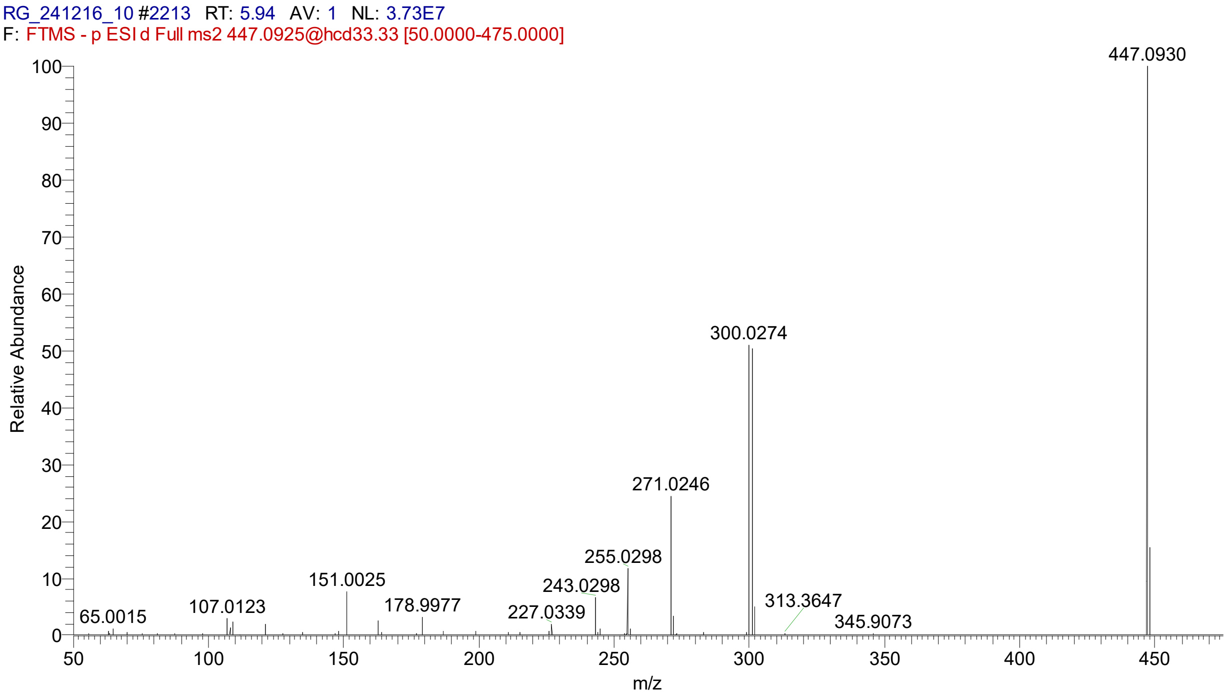

Figure S23. (-) ESI-MS/MS spectrum of quercitrin (**48)** at *m/z* 447.0933 (447.0911-447.0955) (mass accuracy 5 ppm) (for numbers and fragmentation patterns, see Table 1).

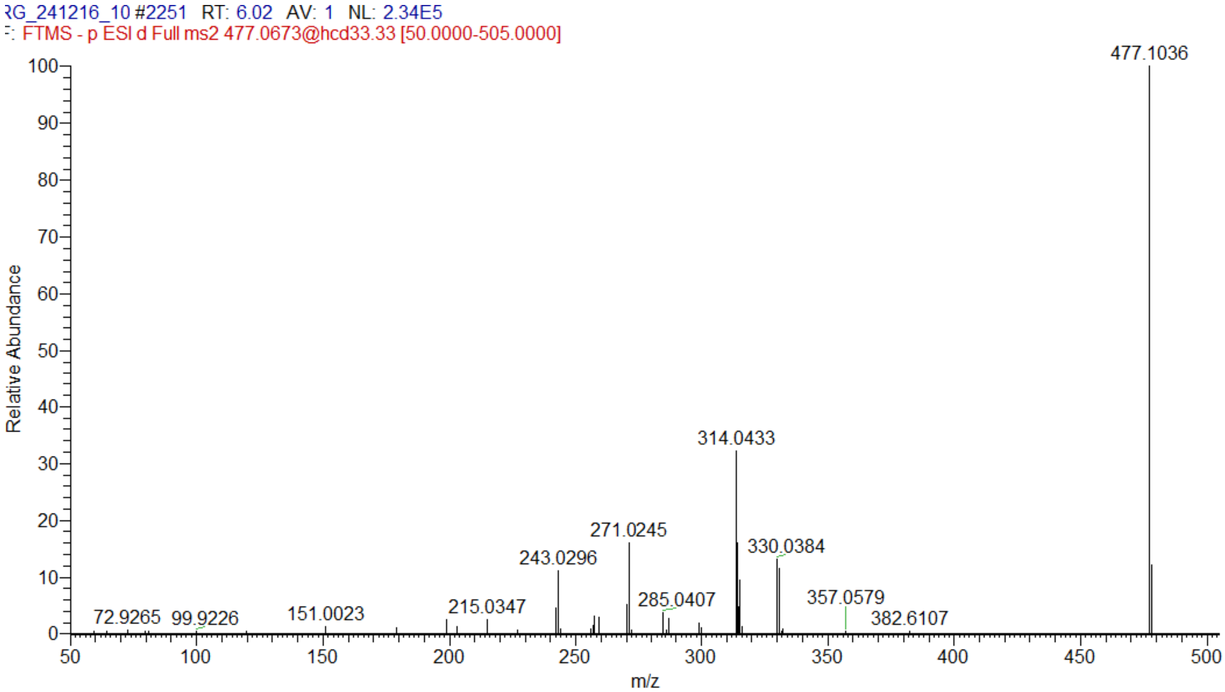

Figure S24. (-) ESI-MS/MS spectrum of isorhamnetin 3-*O*-glucoside (**49)** at *m*/*z* 477.1044 (477.1020-477.1068) (mass accuracy 5 ppm) (for numbers and fragmentation patterns, see Table 1).

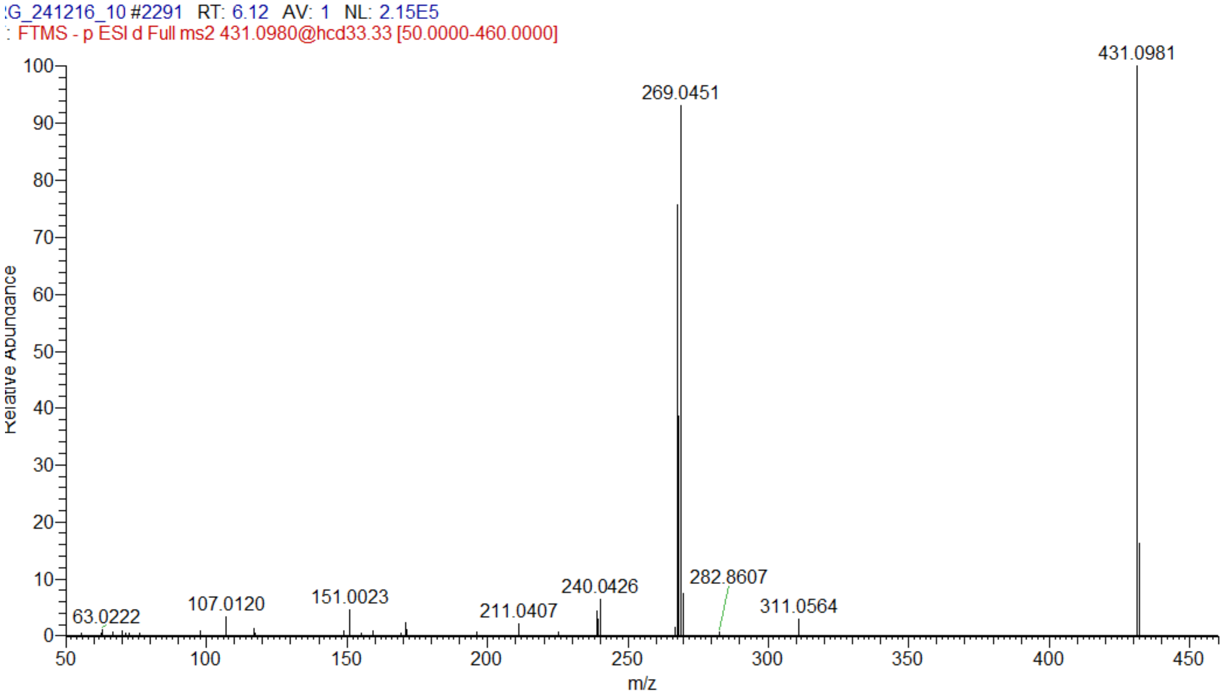

Figure S25. (-) ESI-MS/MS spectrum of apigenin 7-*O*-glucoside (**50)** at *m*/*z* 431.0983 (431.0961-431.1005) (mass accuracy 5 ppm) (for numbers and fragmentation patterns, see Table 1).

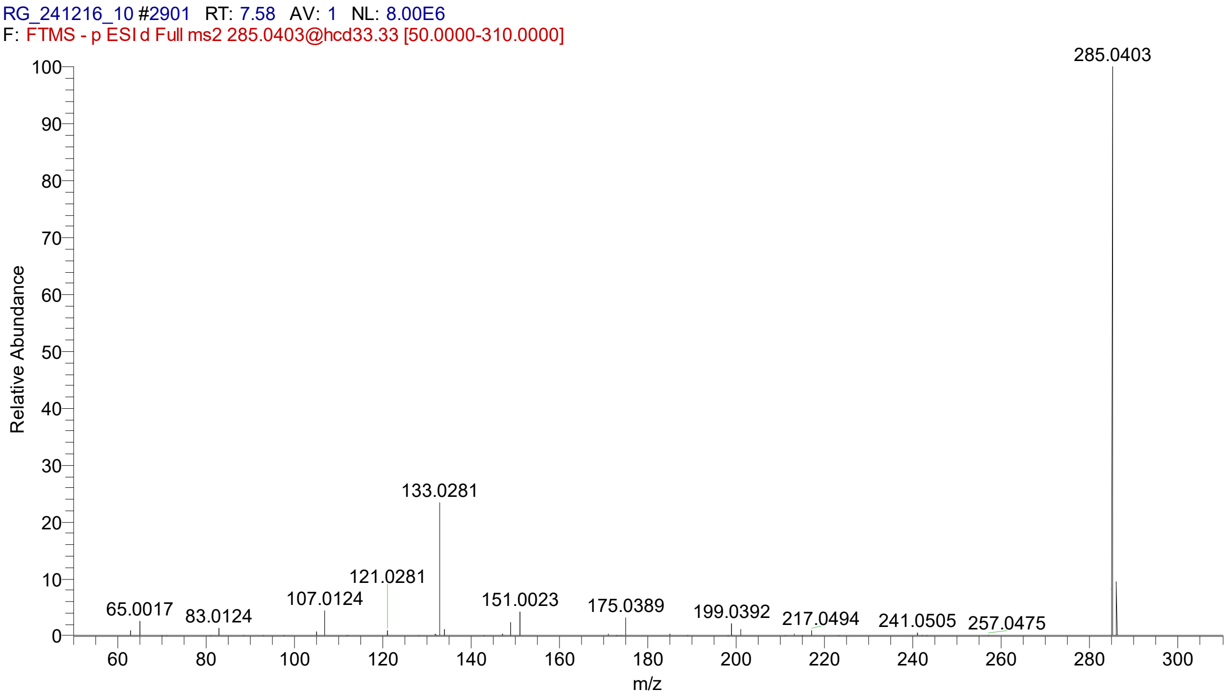

Figure S26. (-) ESI-MS/MS spectrum of luteolin (**52)** at *m/z* 285.0403 (285.0389-285.0417) (mass accuracy 5 ppm) (for numbers and fragmentation patterns, see Table 1).

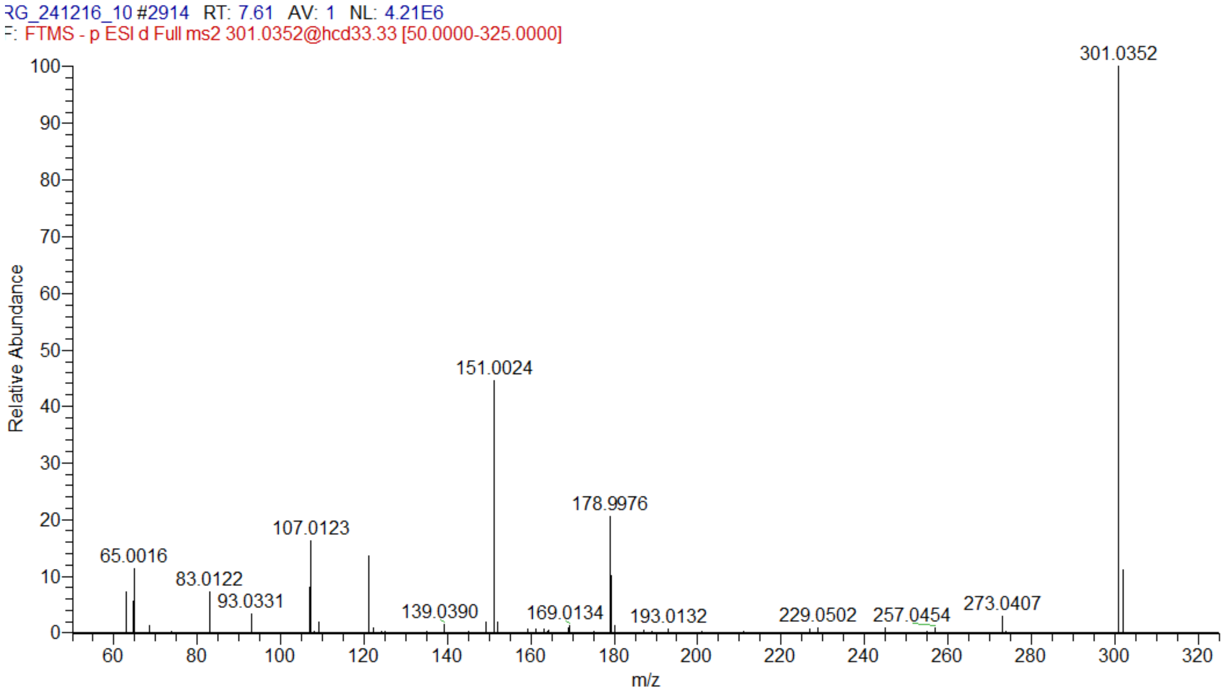

Figure S27. (-) ESI-MS/MS spectrum of quercetin (**53)** at *m/z* 301.0354 (301.0339-301.0369) (mass accuracy 5 ppm) (for numbers and fragmentation patterns, see Table 1).

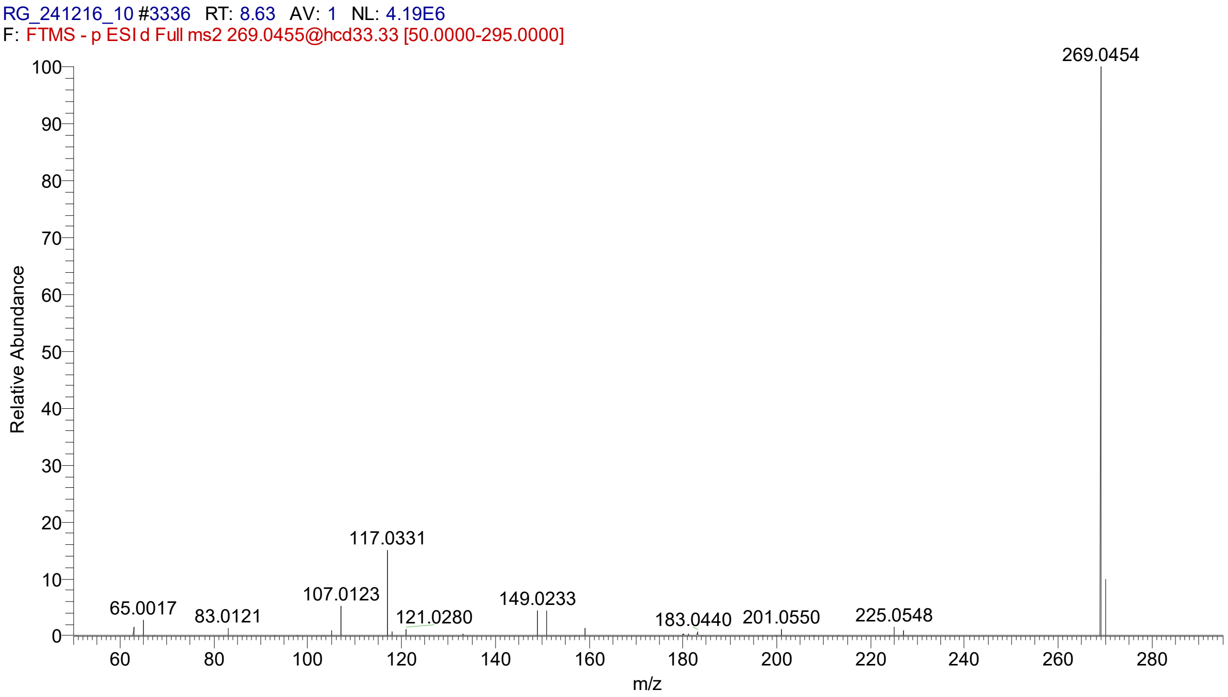

Figure S28. (-) ESI-MS/MS spectrum of apigenin (**54**) at *m/z* 269.0457 (269.0444-269.0470) (mass accuracy 5 ppm) (for numbers and fragmentation patterns, see Table 1).

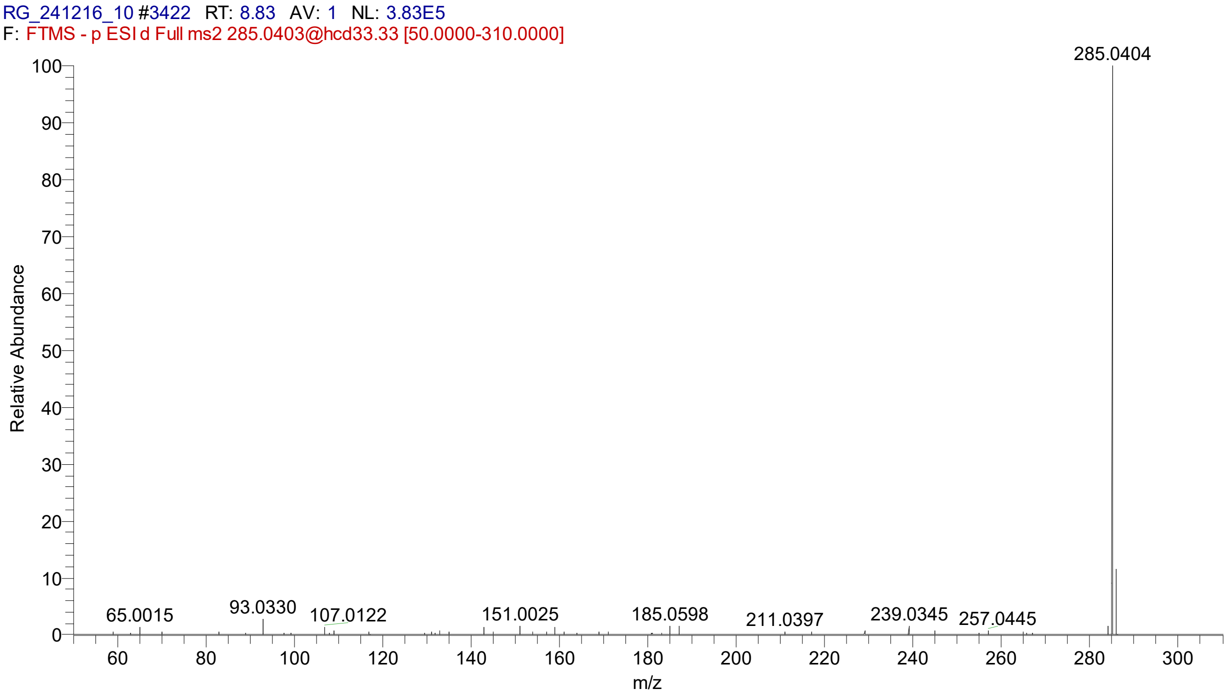

Figure S29. (-) ESI-MS/MS spectrum of kaempferol (**55**) at *m/z* 285.0403 (285.0389-285.0417) (mass accuracy 5 ppm) (for numbers and fragmentation patterns, see Table 1).
